# Frailty affects prognosis in patients with colorectal cancer: A systematic review and meta-analysis

**DOI:** 10.3389/fonc.2022.1017183

**Published:** 2022-11-03

**Authors:** Minghao Cai, Zhongyan Gao, Junyi Liao, Yuanping Jiang, Yong He

**Affiliations:** The Department of Clinical Laboratory Medicine, Yongchuan Hospital of Chongqing Medical University, Chongqing, China

**Keywords:** frailty, colorectal cancer, prognosis, mortality, comorbidities

## Abstract

**Background:**

The prevalence of colorectal cancer has remained high. Most patients have already developed into the middle and advanced stage when they are diagnosed with colorectal cancer, and a small number of them are accompanied by metastasis. In recent years, frailty has been recognized as an important factor affecting the prognosis of colorectal cancer. The aim of this study was to assess the value of frailty on prognosis in patients with colorectal cancer after treatment.

**Method:**

We systematically searched PubMed, Embase, Web Of Science databases up until March2022. A total of 18 studies were retrieved that met the inclusion criteria, including 9 prospective studies and 9 retrospective studies. Frailty screening tools, proportion of frail patients, and outcomes of colorectal cancer patients after treatment were recorded.

**Result:**

18 studies were included with a total of 352,535 participants. Regardless of differences in frailty screening and treatment approaches, outcomes for frailty patients were less favorable in all studies. Compared with the non-frail group, the frail group had higher mortality, more serious complications, more postoperative blood transfusions and delirium, and more support outside the home.

**Conclusion:**

Although there is no uniform standard for frailty screening, assessing the frailty of colorectal cancer patients is of great significance for predicting prognosis of patients after treatment.

## Introduction

Colorectal cancer has become the third most common cancer in the world and the second most deadly cancer in the world (1). It mainly occurs in the elderly, with the highest incidence around the age of 80 (2). Although the standard of therapy for rectal cancer remains surgery with or without neoadjuvant therapy (3), proportion of elderly patients undergoing surgery declines with age due to frailty (4). Frailty is a complex multifactorial syndrome, characterized by a clinically significant increase in vulnerability and worsened health outcomes (5). It affects morbidity and mortality in patients with various cancers (6, 7). Frailty is not only seen in older patients, but younger adults can also fulfil the criteria for frailty (8). Young colorectal cancer patients should also be a group of concern. Cancer patients and those undergoing surgery are more likely to be infirm and have more adverse outcomes than those who are not infirm (9). As a result, oncology societies such as the International Society for Geriatric Oncology (SIOG) recommend frailty screening for older cancer patients (10). Although, some studies have been conducted on frailty and postoperative outcomes and prognosis and a number of frailty screening tools have been invented to assist clinicians in diagnosing (11–13), there is no standard assessment (14). What role does frailty play in the progression of colorectal cancer patients, and What changes have it brought to the prognosis of colorectal cancer patients? There are still many controversies in many studies. We decided to conduct further study on this.

## Methods

### Search strategy

The PubMed, Embase, Web Of Science databases were searched to identify all studies describing frailty and colorectal cancer. The search terms used were related to the following key words: “colon”, “rectum”, “tumor”, “colorectal cancer”, “frailty”. The search string is included in detail in **Appendix A**. The search was completed on April 5, 2022. This study was conducted in accordance with established guidelines [PRISMA (15)].

### Frailty screening tools

The range of frailty tools available to researchers and clinicians can be overwhelming. Due to the diversity of tools, we recommend choosing a frail tool for clinical or research application in cancer patients based on 1) the intent and feasibility of applying the tool to practice and 2) considering specific clinical or research needs, while also taking into account the limitations of available data. The commonly used screening tools are Canadian Study of Health and Aging-Clinical Frailty Scale (CSHA-CFS) (16), American College of Surgeons National Surgical Quality Improvement Program (ACS NSQIP) Modified frailty indices (17, 18), the Edmonton Frail Scale (19), Groningen Frailty Indicator (20), The Kihon Checklist (KCL) (21), Onco-geriatric G8 questionnaire and frailty phenotype (22) and etc.

### Inclusion, exclusion and quality evaluation

We set inclusion and exclusion criteria for prospective and retrospective studies. The degree of frailty must be determined in a clinical setting, and patients were screened for frailty and divided into 2 groups. He specific inclusion and exclusion criteria are as follows.

Inclusion criteria:1. Study type are Case-control studies, cohort studies, cross-sectional studies or RCT;2. Screening of frail patients applies to internationally recognized frailty screening tools;3. Divide patients into frail and non-frail groups for study;4. Primary colorectal cancer without combining other tumors;5. Full text is available, and the data is complete;5. Published publicly, excluding meeting minutes and reviews.

Exclusion criteria:1. non-clinical research;2. Fail to identify or diagnose frailty;3. Patients were not divided into frail and non-frail groups for study;4. non-primary colorectal cancer or colorectal cancer combined with other types of tumors;5. The full text and complete data are not available.

### Assignments

Two independent investigators (J.Y.P and L.J.Y) assessed the studies for eligibility, reached consensus by discussing which studies to include. When two investigators disagreed, a third investigator (C.M.H) was asked to decide on eligibility.

Extracted study data by (C.M.H), including the first author, publication year, study population, study type, sample size of frail patients, frailty assessment tools, and patient quality methods.

Quality assessment was done by two members of the research team (J.Y.P and L.J.Y). They assessed the quality of included studies using the Newcastle-Ottawa Cohort Study Scale. This scale was used to assess 8 questions in three domains. One point is awarded for each satisfactory answer, with a maximum of 9 points. When the score is greater than 5 points, it is considered to be eligible for inclusion. Each study was rated as low (6 points), moderate (7-8 points) or high quality (9 points). If the scores are inconsistent, they will be resolved through negotiation. The evaluation score is shown in **Table 1**.

**Table 1:** Characteristics of studies.

First author, year	Country	Population	Study design	Assessment of frailty	Frail (n)	Type of treatment	Quality Assessment [Newcastle-Ottawa Scale (NOS)]
A. Aaldriks,2013	Netherlands	N=143 41% female Mean age of 75 (range 70-92 years)	prospective study	Groningen Frailty Indicator (GFI)	34	Chemotherapy (Adjuvant Chemotherapy,n=54,54%female; Palliative Chemotherapy,n=89,34%female)	7
A. AL-Khamis,2019	USA	N=295490\52.5% female Age≥18 years (45.4% ≥65 years) 72.7% White 9.1% Black 2.5% Asian	retrospective study	Five-item modified frailty index (5-mFI)	53230	elective or non-elective colorectal procedure(exclude emergency procedure)	9
Giacomo Pata,2020	Italy	N=104 47% female The median age was 81 years (range 75–95 years)	prospective multicentric cohort study	The Multidimensional Prognostic Index (MPI; Pilotto et al)	34	colorectal cancer surgery	8
Hirohisa Okabe,2018	Japan	N=269 38% female Age≥65 years	retrospective study	Clinical Frailty Scale (CFS)(Rockwood K et al. A global clinical measure offitness and frailty in elderly people)	78	elective colorectal surgery(palliative procedures were excluded from the study)	8
Koichi Tamura,2021	Japan	N=500 41.8% female Median age was 76 years(range 65-96 years) 10 patients≥90 years	prospective study	The Kihon Checklist (KCL) (directed by the Japanese Ministry of Health, Labor and Welfare)	164	elective colorectal surgery	7
Kosuke Mima,2020	Japan	N=729 47% female Age≥18 years (46% ≥75 years)	retrospective study	Clinical Frailty Scale (CFS)(Rockwood K et al. A global clinical measure offitness and frailty in elderly people)	253	curative resection of colorectal cancer	7
Manuel Artiles-Armas,2021	Spain	N=149 35.6% female Median age was 75 years (range 72–80 years)	prospective cohort study	The Canadian Study of Health and Aging-Clinical Frailty Scale (CSHA-CFS)	59	elective colorectal surgery	9
NINA OMMUNDSEN,2014	Norway	N=178 57% female Age≥70 years (6% ≥90 years)	prospective study	Geriatric assessment (GA)(Ellis G et al,Comprehensive geriatric assessment for older adults admitted to hospital,2011)	76	elective surgery	7
Simon J. G. Richards,2020	New Zealand	N=86 50% female Median age was 76 years (range 72–81 years)	prospective observational study	The Edmonton Frail Scale (EFS)	12	elective colorectal cancer surgery	8
Stan A.M. Bessems,2020	Netherlands	N=132 44% female Median age was 78 years (range 70–90 years)	retrospective observational study	the Geriatric-8 (G8) and the 4-m gait speed test (4MGST)	53	elective colorectal cancer surgery	8
T.E. Argillander,2022	Netherlands	N=231 55% female Median age was 76 years (range 73–81 years)	retrospective cohort study	Groningen Frailty Indicator (GFI)	44	colorectal cancer (CRC) surgery	8
Wenbin Gong,2018	China	N=241 46.5% female The mean age was 68.4 years (SD 11.7)	retrospective study	the Modified Frailty Index (mFI) (derived from the Canadian Study of Health and Aging Frailty Index)	19 (mFI:Intermediate,n=81;low,n=141)	elective colorectal cancer resections(Emergency cases and non-primary tumor resections were excluded)	7
K. Beukers,2021	Netherlands	N=97 51.5% female The mean age was 77.2 years (SD 4.8)	retrospective multicentre study	the Geriatric-8 (G8)	49	adjuvant chemotherapy	6
Susanna Niemeläinen,2021	Finland	N=161 60% female The mean age was 84.5 years (range 80-97 years)	prospective, multicentre observational study	Clinical Frailty Scale (CFS)(Rockwood K et al. A global clinical measure offitness and frailty in elderly people)	43	elective colon cancer surgery	7
Viraj Pandit,2018	USA	N=53652 38% female The mean age was 69 years (SD 19) 40% White	retrospective study	CCFI(Seven variables assessed in the Canadian Study of Health and Aging Frailty Index (CSHA-FI) were matched to preoperative variables collected in the NIS database)	18241	elective colon cancer surgery(excluded patients who underwent emergent surgery or had rectal cancer)	8
Esteban T.D. Souwer,2017	Netherlands	N=139 45% female The mean age was 77.7 years (range 75.0–82.8 years)	prospective cohort study	the Geriatric 8 (G8) and Identification of Seniors at Risk for Hospitalized Patients (ISAR-HP)	20	colorectal cancer surgery(exclude emergency surgery , Transanal Endoscopic Microsurgery, stage IV disease and synchronous cancer at time of diagnosis)	8
Elizabeth M. Cespedes Feliciano,2020	USA	N=126 100% female	multicenter, prospective cohort study	a frailty score( defined in Woods NF et al 2005;Fried LP et al 2001;Erratum.J Am Geriatr Soc. 2017)	78	Not mentioned	7
Toshihiro Nakao,2021	Japan	N=108 33.3% female Median age was 70 years (range 42–93 years)	retrospective study	Clinical Frailty Scale (CFS)(Rockwood K et al. A global clinical measure offitness and frailty in elderly people)	11	colorectal cancer radical surgery	8

### Research indicators

The indicators we observed were the outcomes of colorectal cancer patients (frail and non-frail groups) after treatment. The primary outcome measure was mortality and complication rate, and the secondary outcome measures were delirium, postoperative blood transfusion, discharge destination other than home, readmission, and length of hospital stay.

### Statistical analysis

We extracted data from all publications to calculate standard mean difference (SMD) and associated 95% confidence interval (CI) for continuous outcomes. P<0.05 was considered statistically significant. The presence of statistical heterogeneity of the results was assessed by using the I^2^ measure, with I^2^ >50% considered significant when P ≤ 0.10. If there was no heterogeneity (P value for heterogeneity > 0.1), a fixed-effects model was chosen to calculate ensemble effects; otherwise, a random-effects model was used. We performed a meta-analysis of all studies, and Subgroup analyses were also performed for mortality and complication classification.

STATA15.1 software was used for the standard meta−analysis and the sensitivity analysis.

## Result

**Figure 1:**
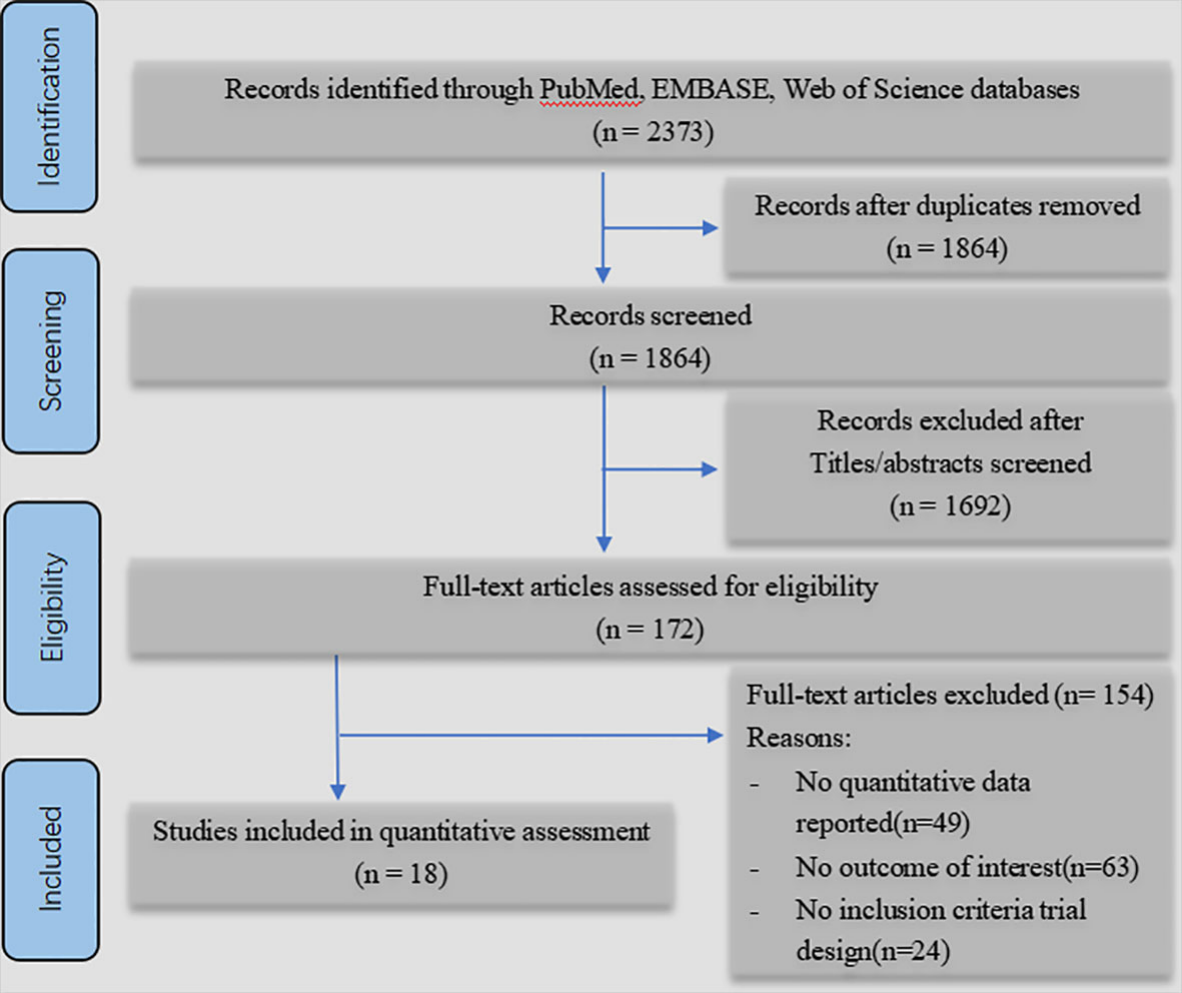
PRISMA flow diagram.

After initial screening of PubMed, Embase and Web Of Science databases, a total of 2373 studies were identified: 704 from Web Of Science, 701 from PubMed, and 968 from Embase. We performed title/abstract screening and full-text reading after adding constraints such as publication year, repetition, full-text reviews, and cross-references. Finally, 18 studies and 352,535 patients were included in this meta-analysis. Of the 18 studies, 9 were from Europe, 3 from North America, 5 from Asia, and 1 from Oceania. All included subjects were over 18 years old, and mainly consisted of the elderly over 65 years old. The ratio of males and females is relatively equal. The retrieval process is shown in **Figure 1**.

Among the 18 studies, retrospective studies and prospective studies each accounted for 9. Mortality was assessed in 12 studies. Complications was assessed in 12 studies. Delirium was assessed in 3 studies. Postoperative blood transfusion was assessed in 3 studies. Discharge destination not home (nursing facility or other) was assessed in 4 studies. Readmission was assessed in 4 studies and hospital stay was assessed in 9 studies. Among them, mortality (30-day, 90-day, 1-year, 2-year, 5-year mortality) and complications (according to Clavien–Dindo grade (23) 1-2 for minor, ≥3 for severe) were evaluated in subgroup analysis.

Each study assessed frailty differently, and the tools used to assess frailty are shown in **Table 1**.

These studies have an average score of 7.6 in the quality evaluation. The full score is 9 points. All studies fulfilled the inclusion criteria.

### Frailty and mortality

**Figure 2:**
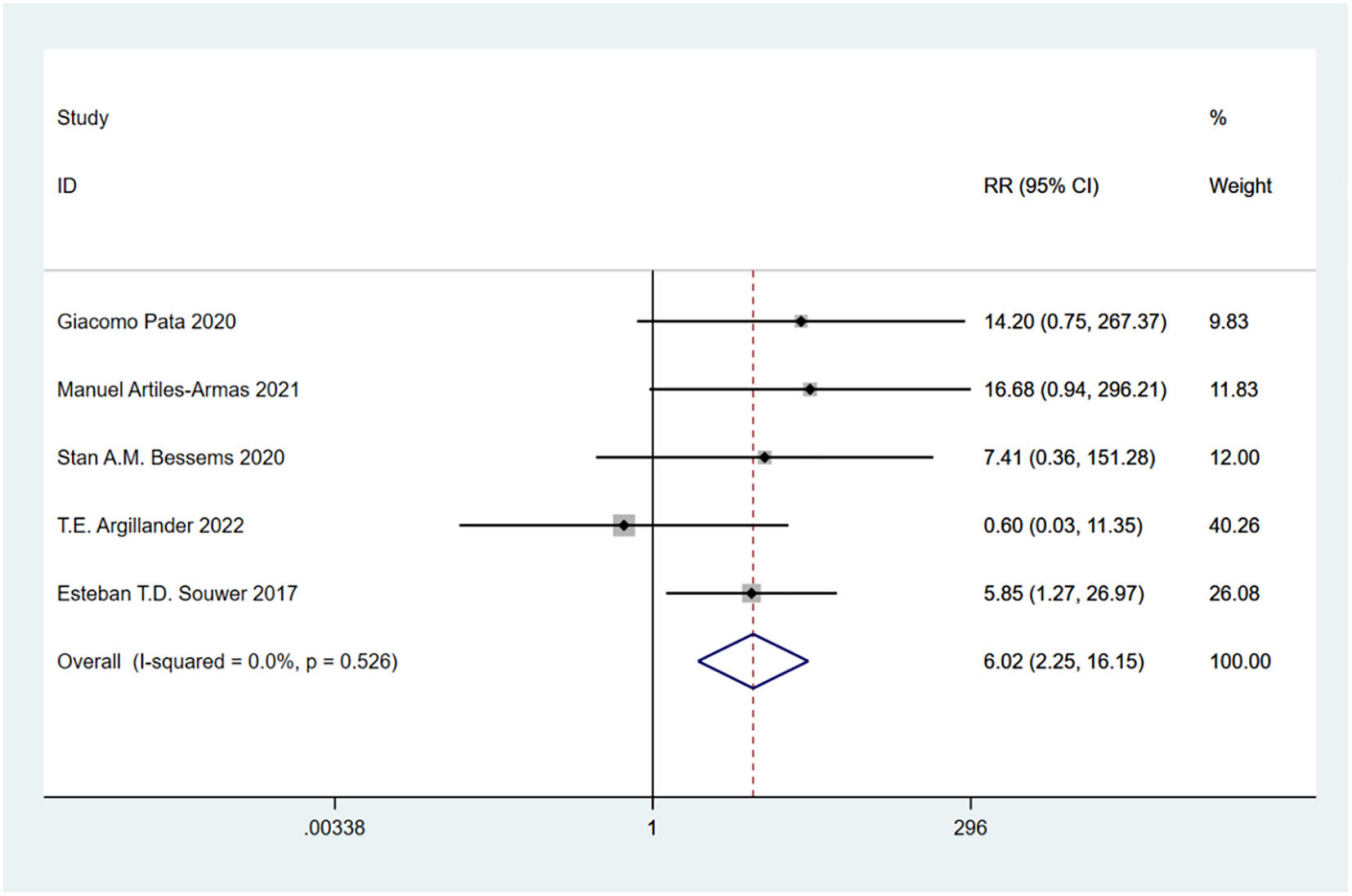
30-day mortality.

**Figure 3:**
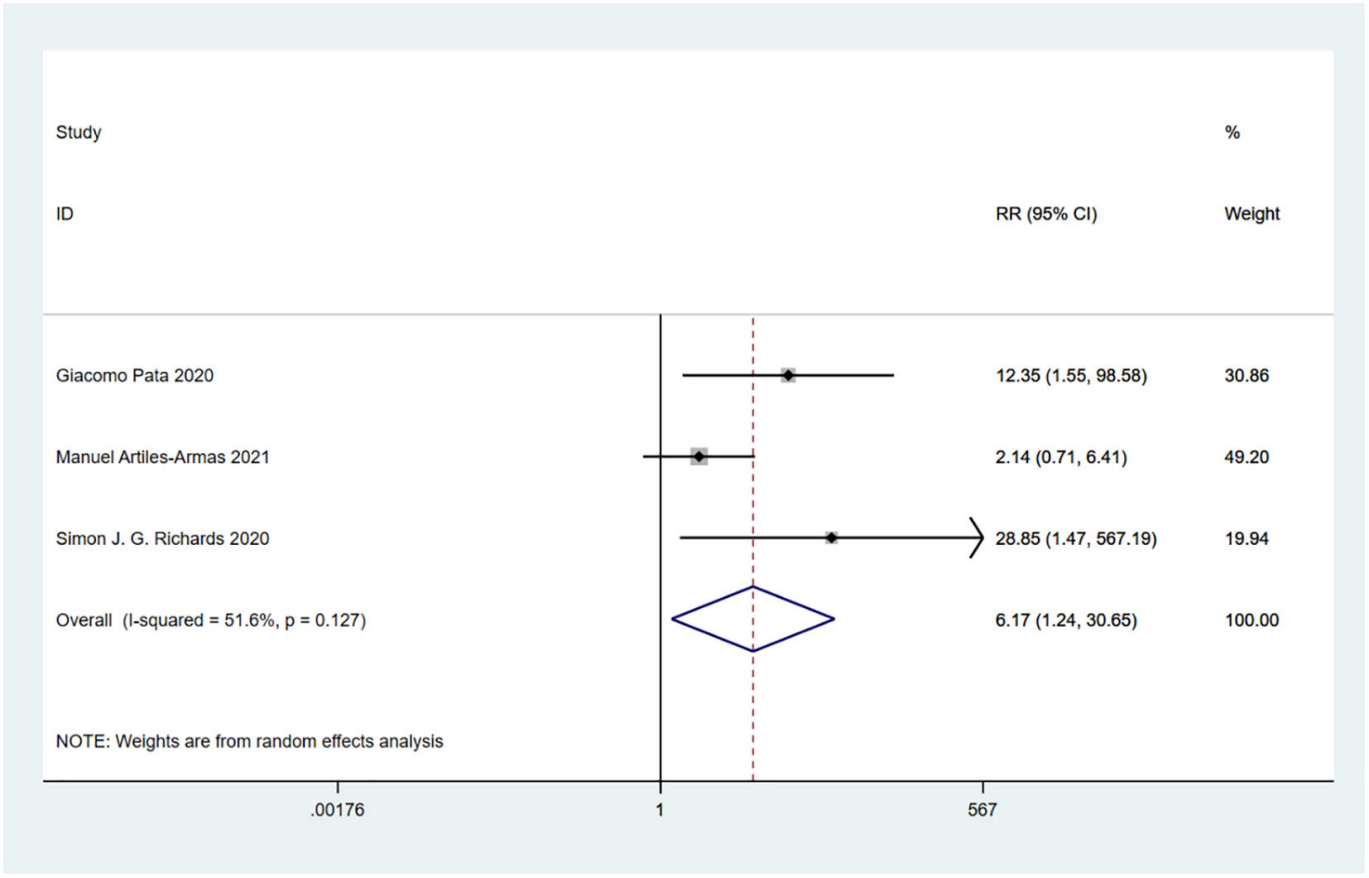
90-day mortality.

**Figure 4:**
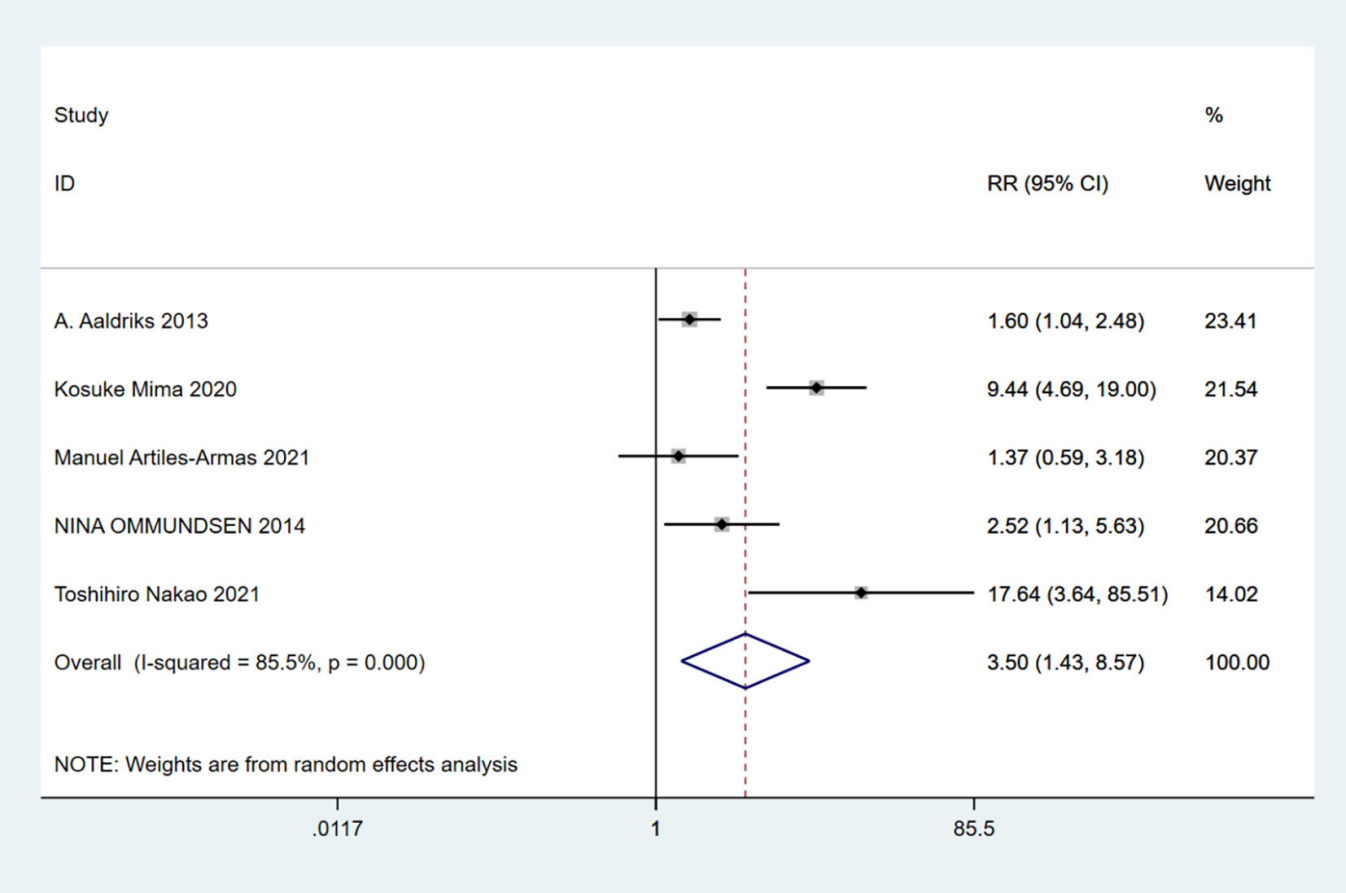
1-year mortality.

**Figure 5:**
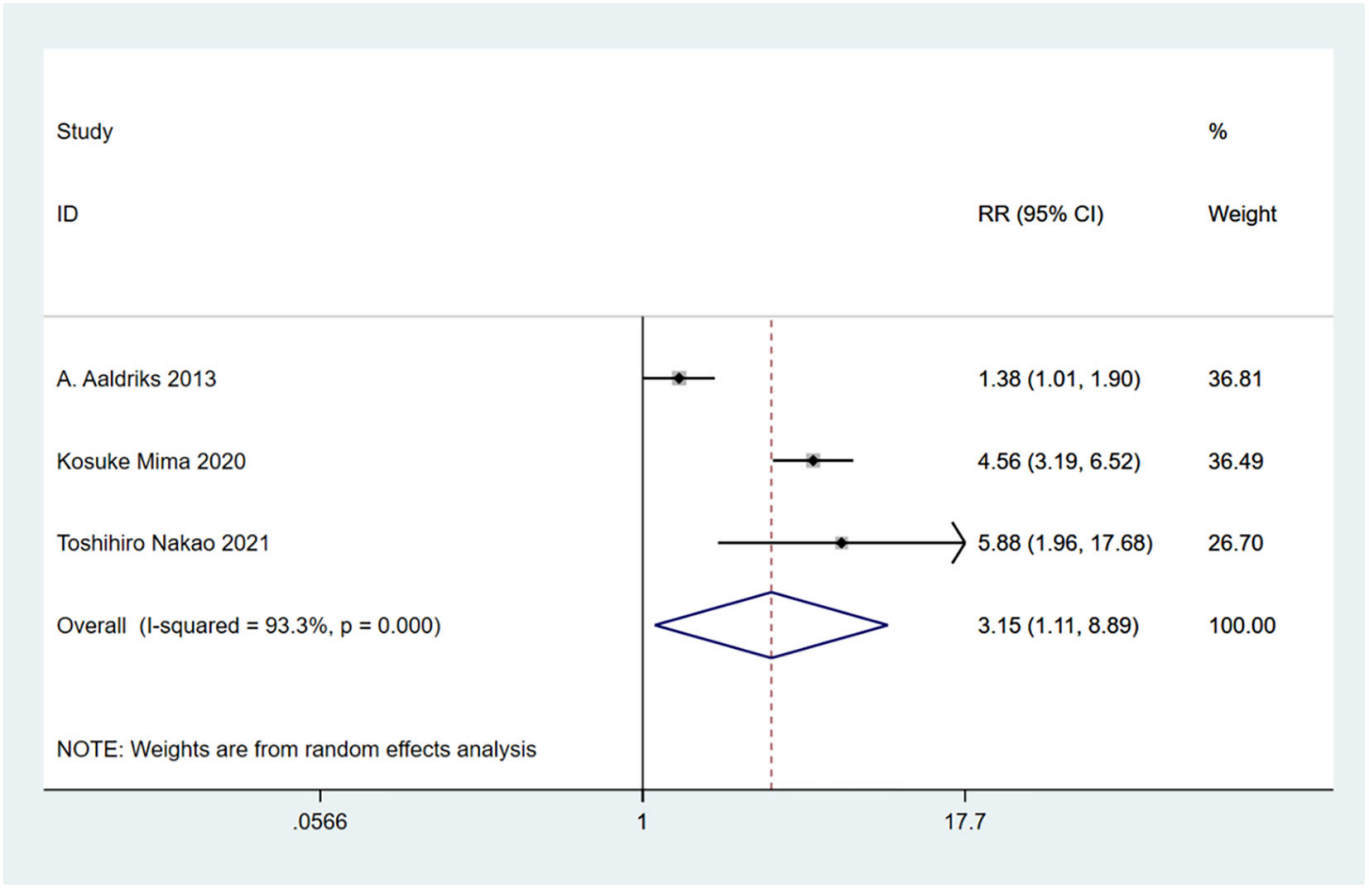
2-year mortality.

**Figure 6:**
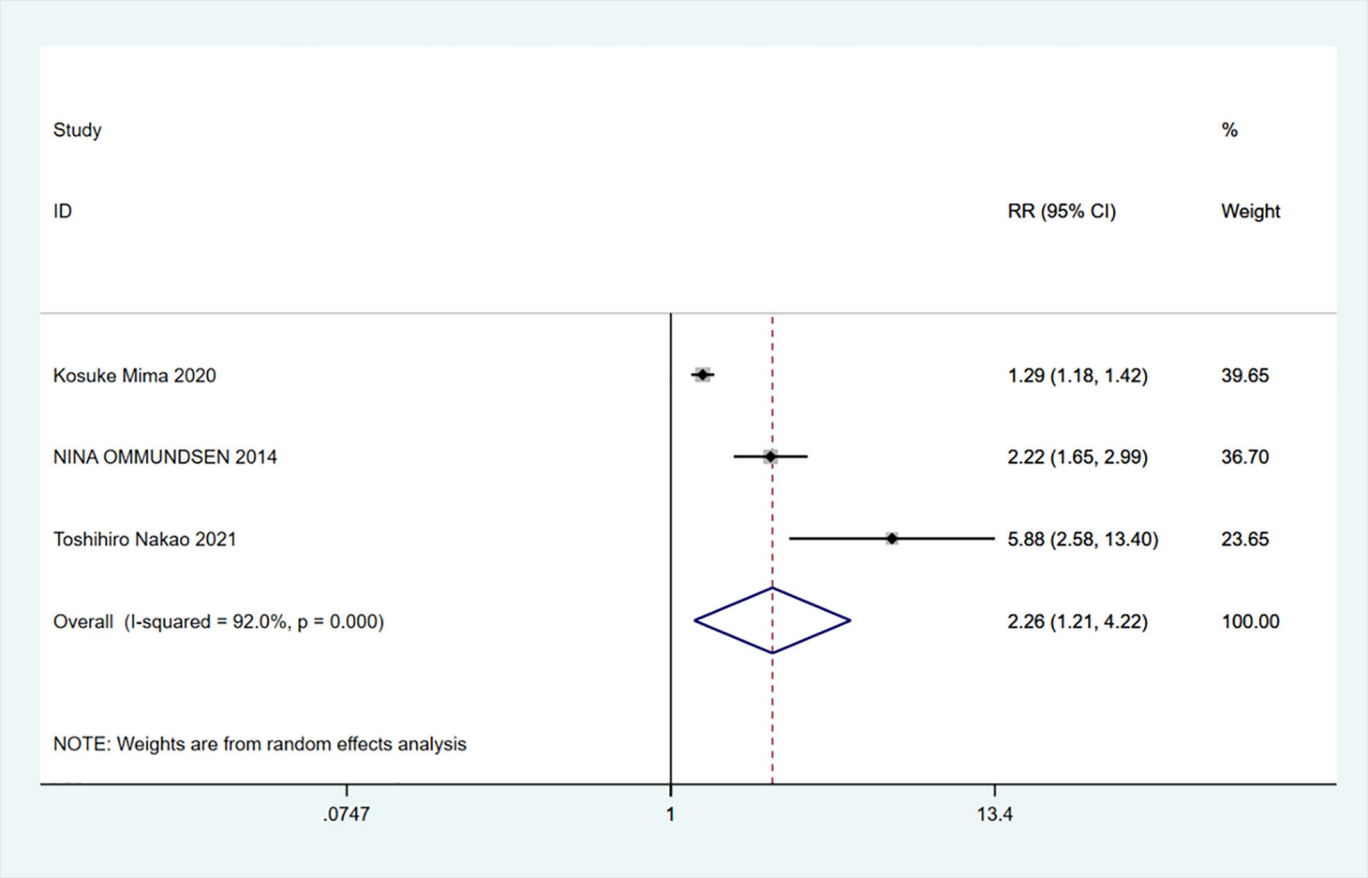
5-year mortality.

A total of 10 studies involving 4,721 patients were included. Heterogeneity test was performed. The group of 30-day mortality: I-squared=0.0%, p=0.526, the heterogeneity was not significant, a fixed effects model was adopted, RR (95%CI) =6.02 (2.25, 16.15). The difference was statistically significant between the frail group and the non-frail group; The group of 90-day mortality: I-squared=51.6%, p=0.127, the heterogeneity was significant, a random effects model was adopted, RR (95%CI) =6.17 (1.24, 30.65). The difference was statistically significant between the frail group and the non-frail group; The group of 1-year mortality: I-squared=85.5%, p=0, the heterogeneity was significant, a random effects model was adopted, RR (95%CI) =3.50 (1.43, 8.57). The difference was statistically significant between the frail group and the non-frail group; The group of 2-year mortality: I-squared=93.3%, p=0, the heterogeneity was significant, a random effects model was adopted, RR (95% CI) =3.15 (1.11, 8.89). The difference was statistically significant between the frail group and the non-frail group; The group of 5-year mortality: I-squared=92.0%, p=0, the heterogeneity was significant, a random effects model was adopted, RR (95%CI) =2.26 (1.21, 4.22), The difference was statistically significant between the frail group and the non-frail group. Thus, frailty was associated with increased mortality in patients with colorectal cancer after treatment (**Figures 2**–**6**).

### Frailty and complications

**Figure 7:**
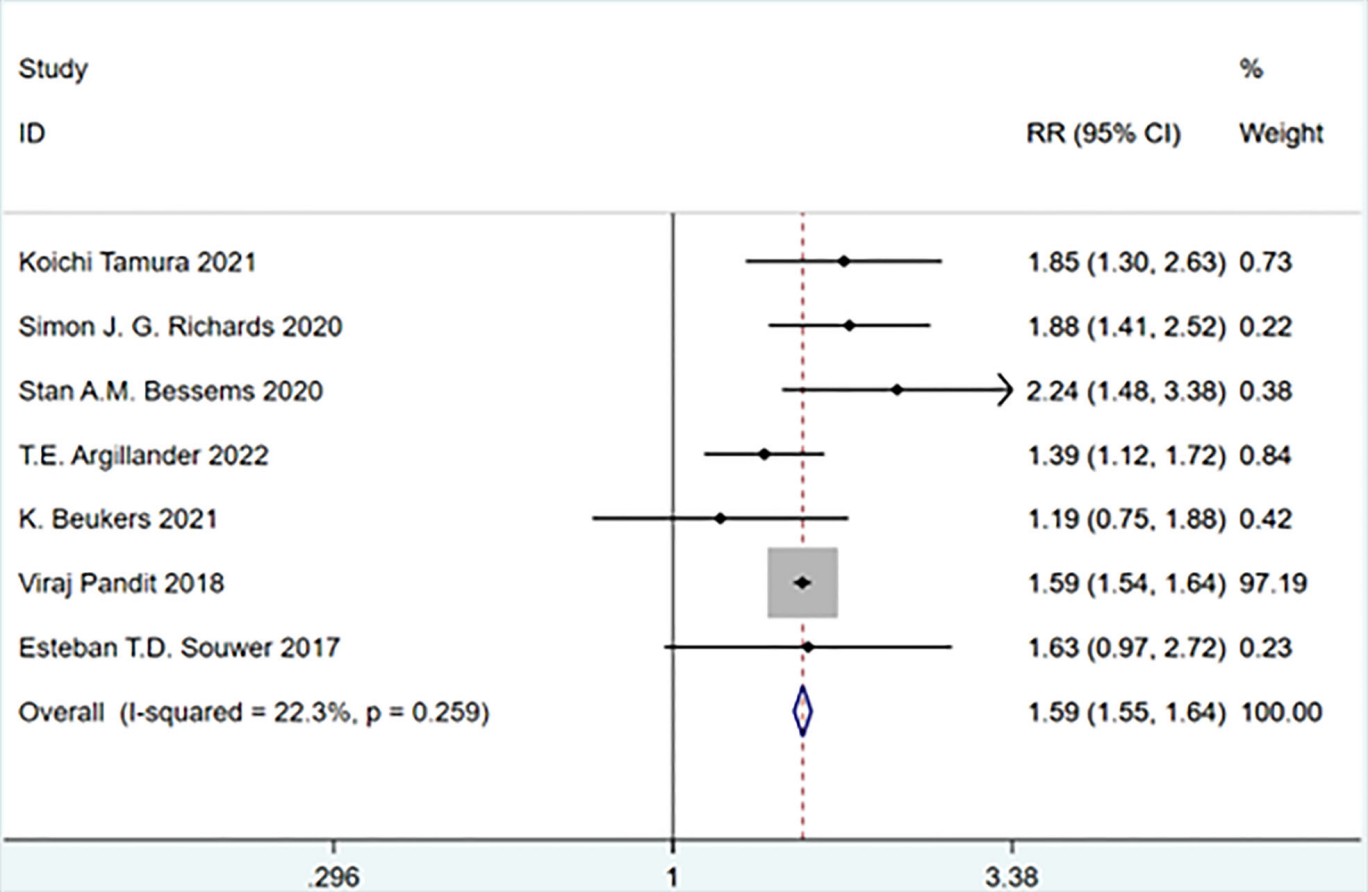
Total complications.

**Figure 8:**
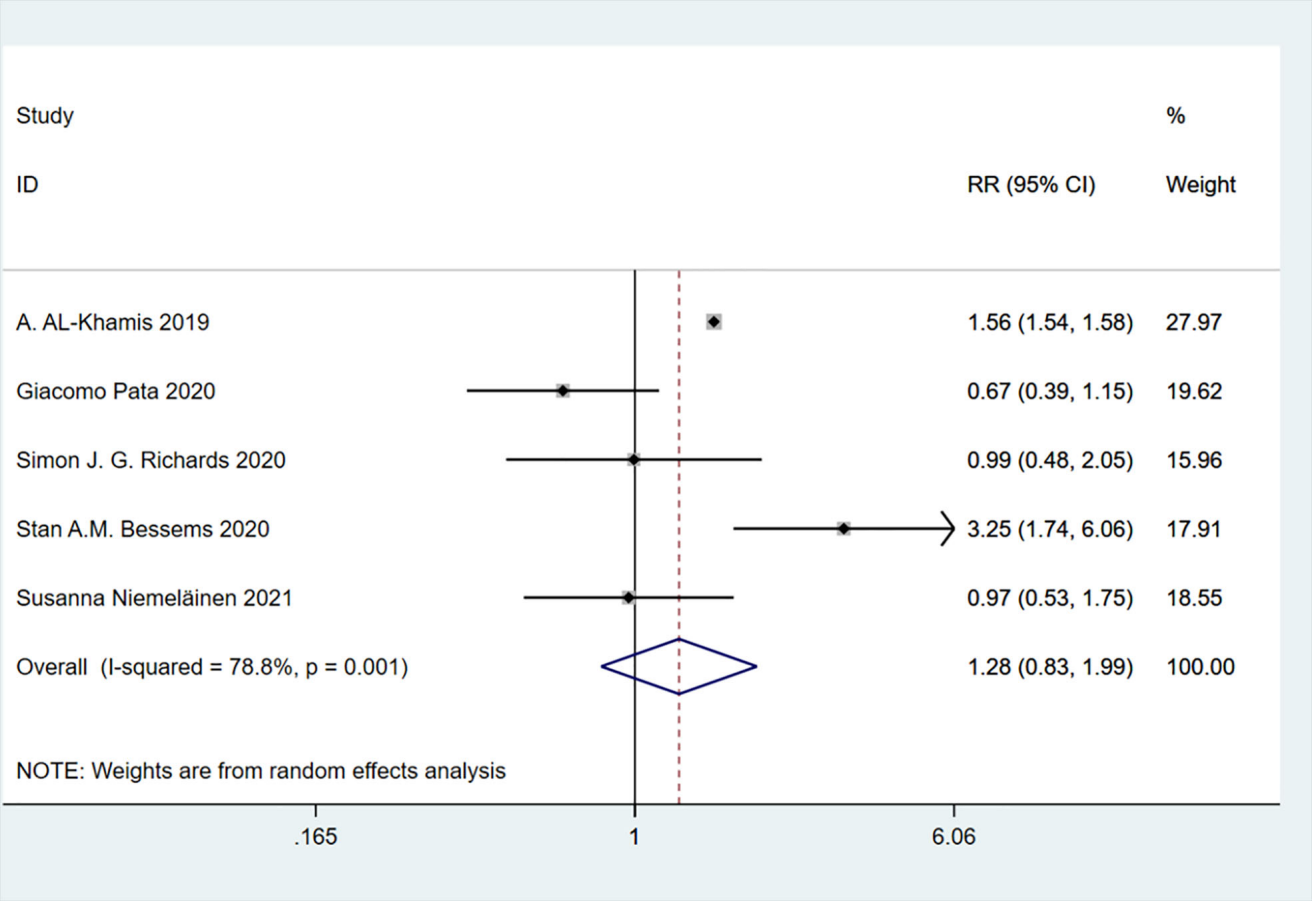
Minor complications.

**Figure 9:**
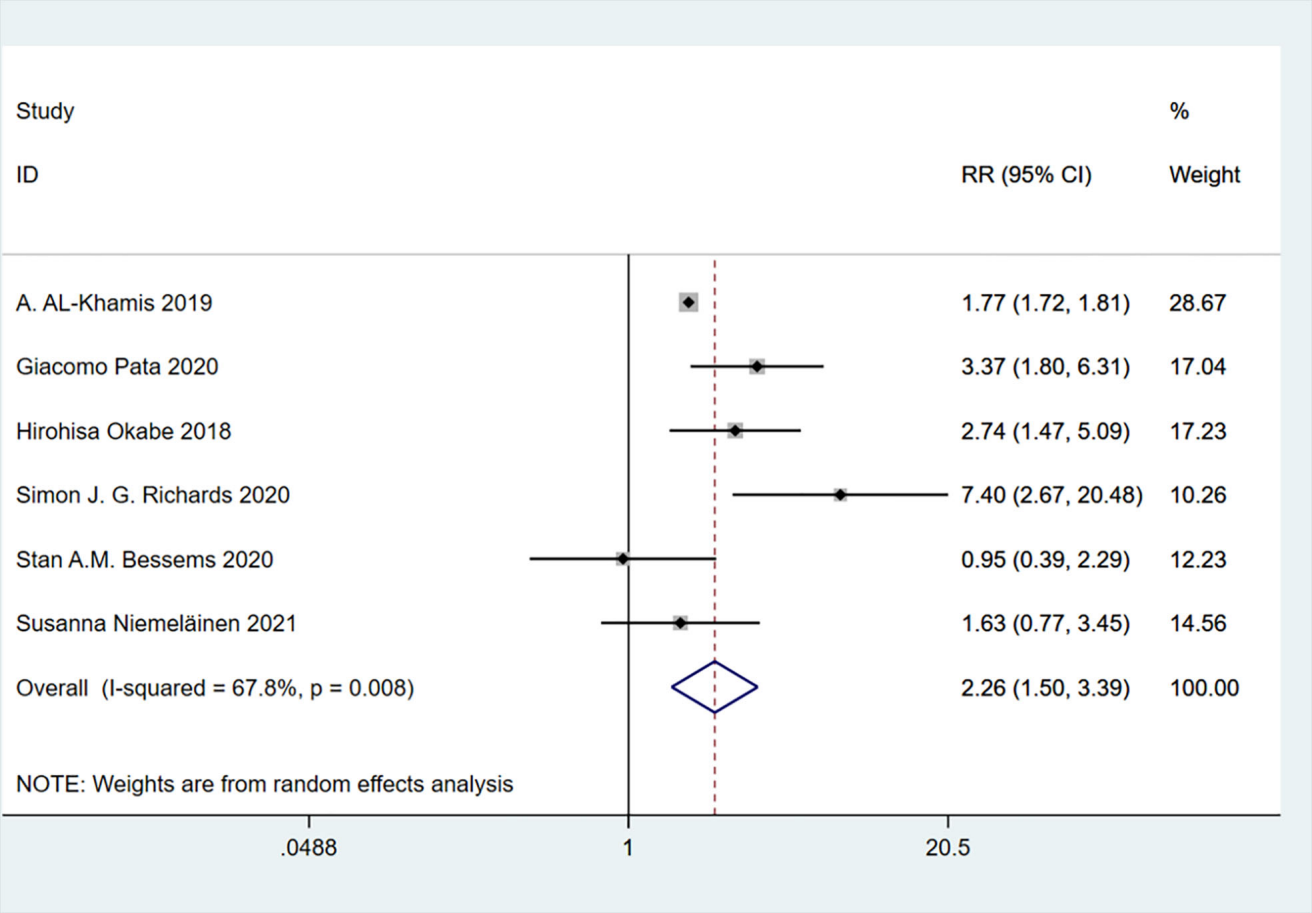
Severe complications.

A total of 7 studies involving 54,835 patients were included. Heterogeneity test was performed. The group of total complications: I-squared=22.3%, p=0.259, the heterogeneity was not significant, a fixed effects model was adopted, RR (95%CI) =1.59 (1.55, 1.64). The difference was statistically significant between the frail group and the non-frail group; The group of minor complications: I-squared=78.8%, p=0.001, the heterogeneity was significant, a random effects model was adopted, RR (95%CI) =1.28 (0.83, 1.99). There was no significant difference between the frail group and the non-frail group; The group of severe complications: I-squared=67.8%, p=0.008, the heterogeneity was significant, a random effects model was adopted, RR (95%CI) =2.26 (1.50, 3.39). The difference was statistically significant between the frail group and the non-frail group. We found that frailty did not appear to have a significant effect on minor complications after treatment but had a significant effect on severe complications (**Figures 7**–**9**).

### Frailty and delirium

**Figure 10:**
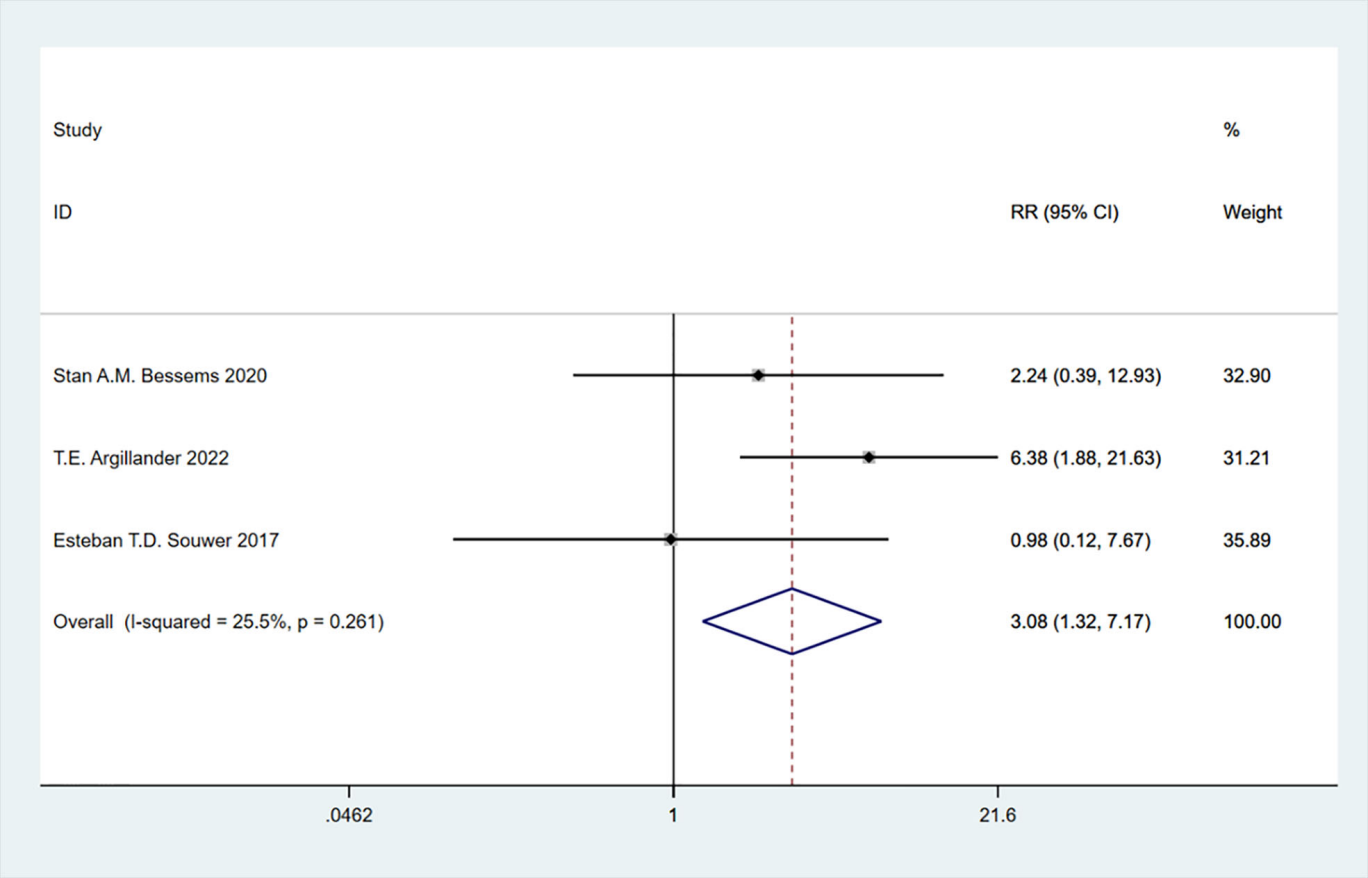
Delirium.

A total of 3 studies involving 500 patients were included. Heterogeneity test was performed. The group of delirium: I-squared=25.5%, p=0.261, the heterogeneity was not significant, a fixed effects model was adopted, RR (95%CI) =3.08 (1.32, 7.17). The difference was statistically significant between the frail group and the non-frail group. It can be discovered that frailty may be associated with high incidence of delirium (**Figure 10**).

### Frailty and postoperative blood transfusion

**Figure 11:**
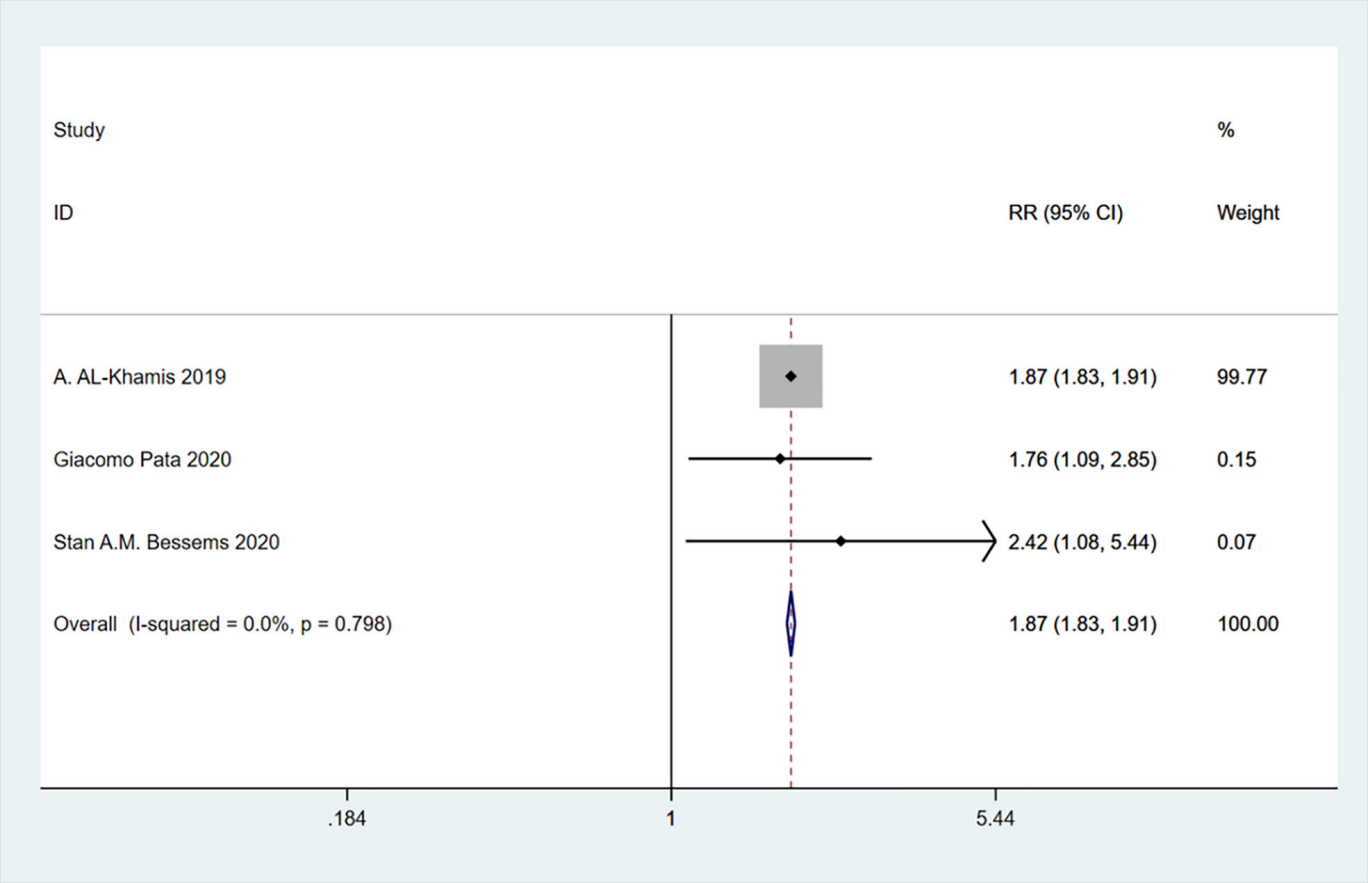
Postoperative blood transfusion.

A total of 3 studies involving 295,724 patients were included. Heterogeneity test was performed. The group of postoperative blood transfusion: I-squared=0.0%, p=0.798, the heterogeneity was not significant, a fixed effects model was adopted, RR (95%CI) =1.87 (1.83, 1.91). The difference was statistically significant between the frail group and the non-frail group. We can find that frailty may be associated with high likelihood of postoperative blood transfusion (**Figure 11**).

### Frailty and discharge destination not home

**Figure 12:**
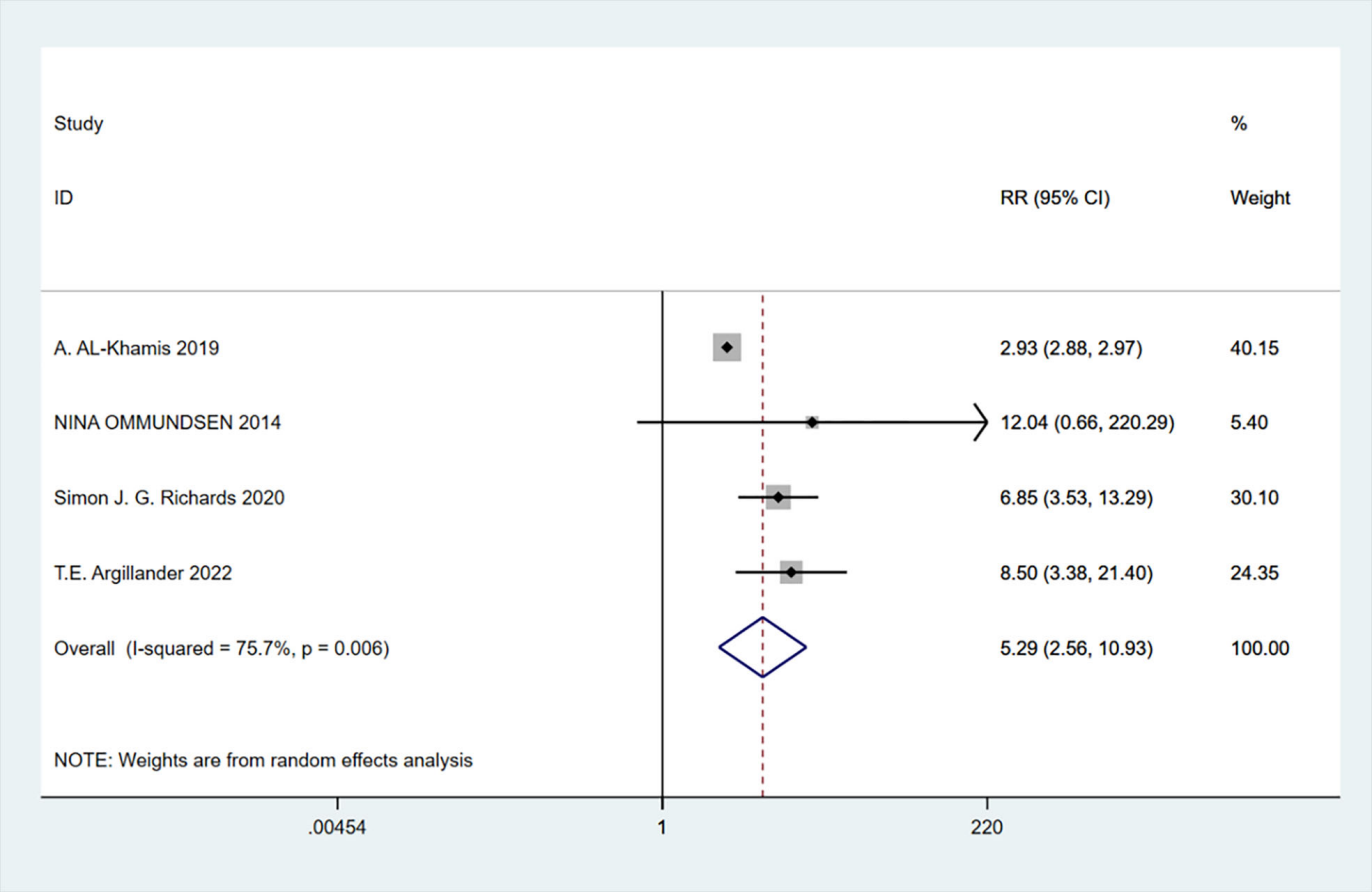
Discharge destination not home.

A total of 4 studies involving 295,983 patients were included. Heterogeneity test was performed. The group of discharge destination not home: I-squared=75.7%, p=0.006, the heterogeneity was significant, a random effects model was adopted, RR (95%CI) =5.29 (2.56, 10.93). The difference was statistically significant between the frail group and the non-frail group. The frail group could be found to have a higher risk of readmission (**Figure 12**).

### Frailty and readmission

**Figure 13:**
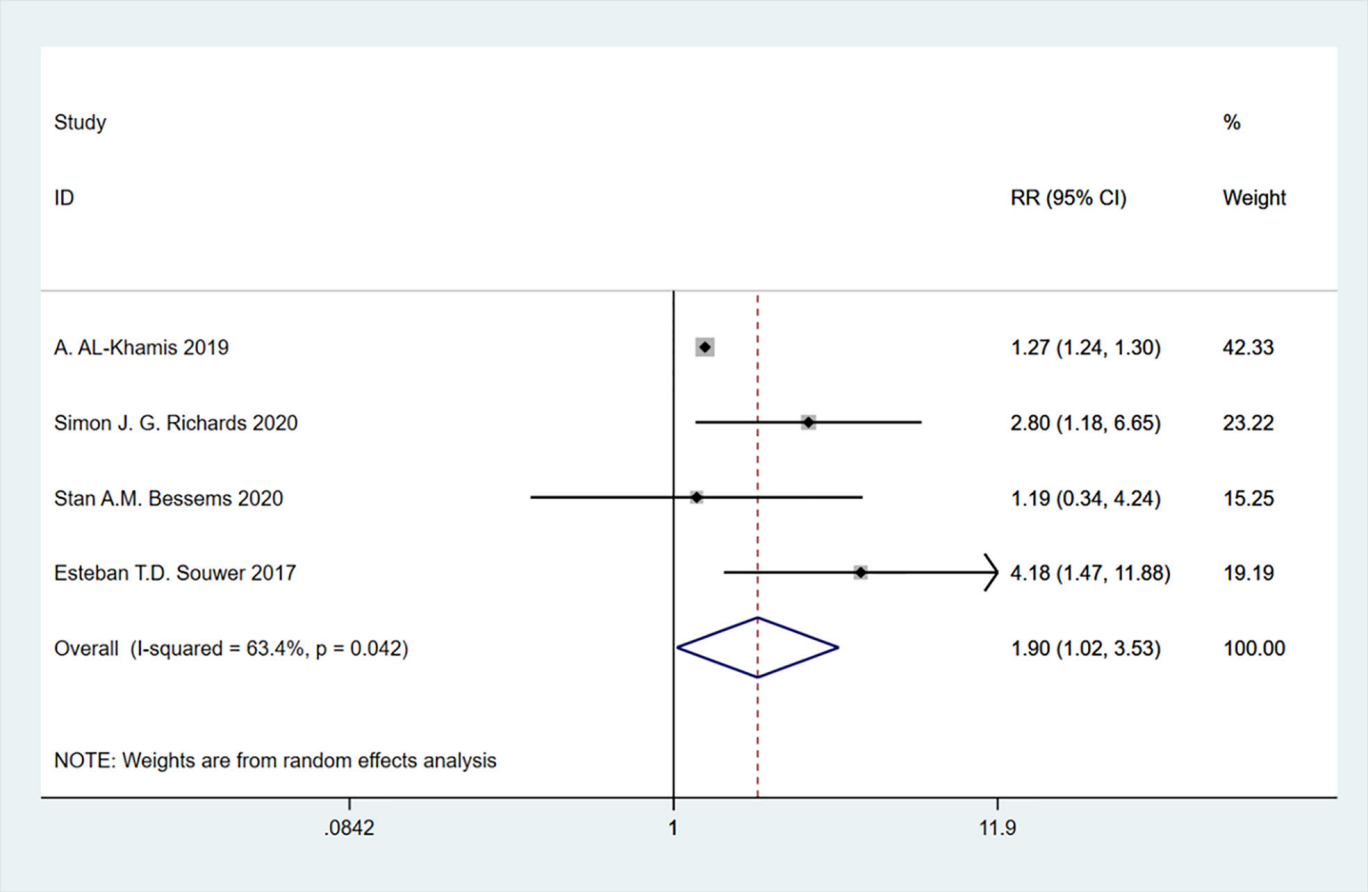
Readmission.

A total of 4 studies involving 295,843 patients were included. Heterogeneity test was performed. The group of readmissions: I-squared=63.4%, p=0.042, the heterogeneity was significant, a random effects model was adopted, RR (95%CI) =1.90 (1.02, 3.53). The difference was statistically significant between the frail group and the non-frail group. The frail group could be found to have a higher risk of readmission (**Figure 13**).

### Frailty and hospital stay

**Figure 14:**
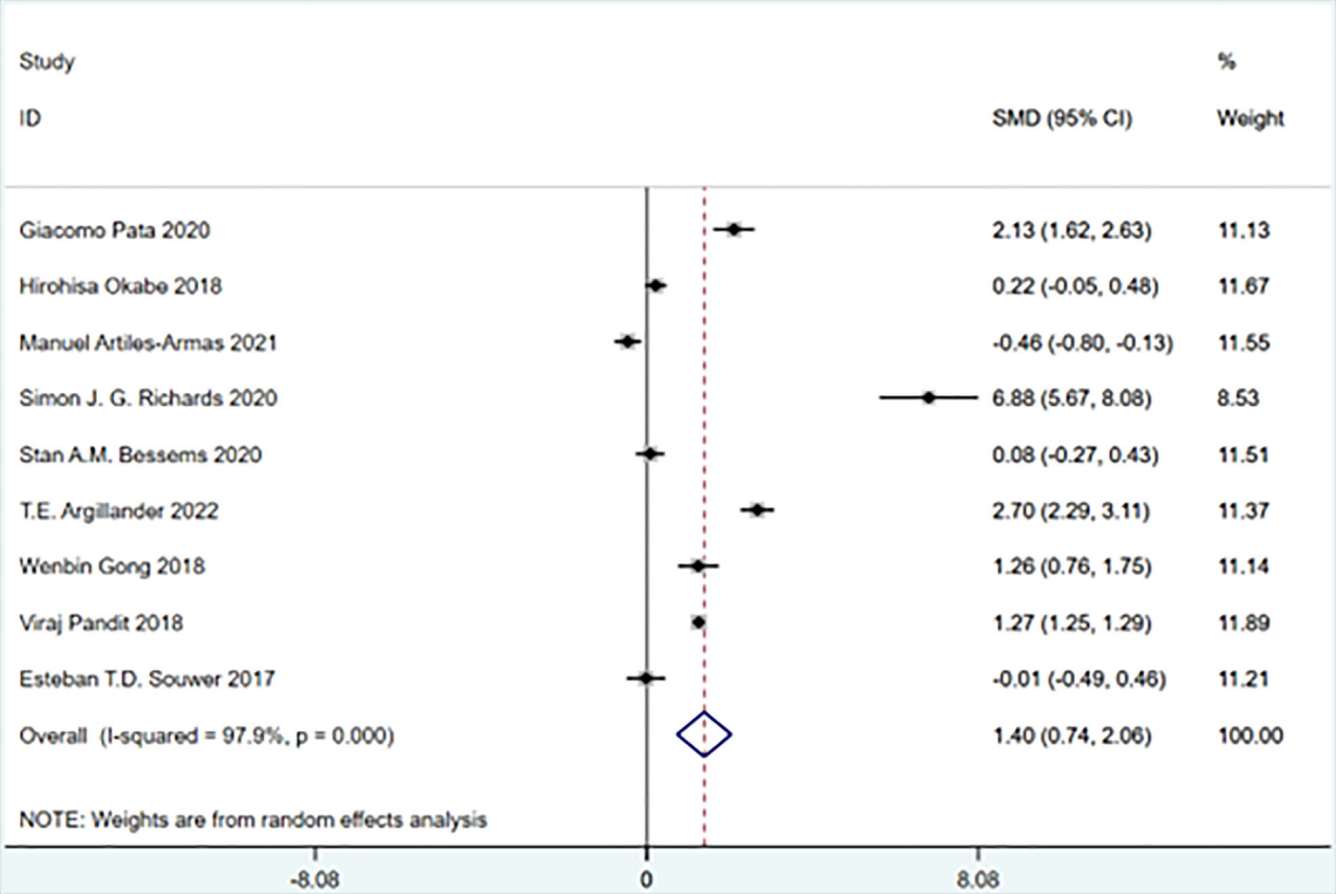
Hospital stay.

A total of 9 studies involving 54,920 patients were included. Heterogeneity test was performed. The group of hospital stay: I-squared=97.9%, p=0.000, the heterogeneity was significant, a random effects model was adopted, RR (95%CI) =1.40 (0.74, 2.06). The difference was statistically significant between the frail group and the non-frail group. The frail group could be found to have a higher risk of readmission (**Figure 14**).

### Subgroup analysis

#### Frailty and mortality at different follow-up times

**Figure 15:**
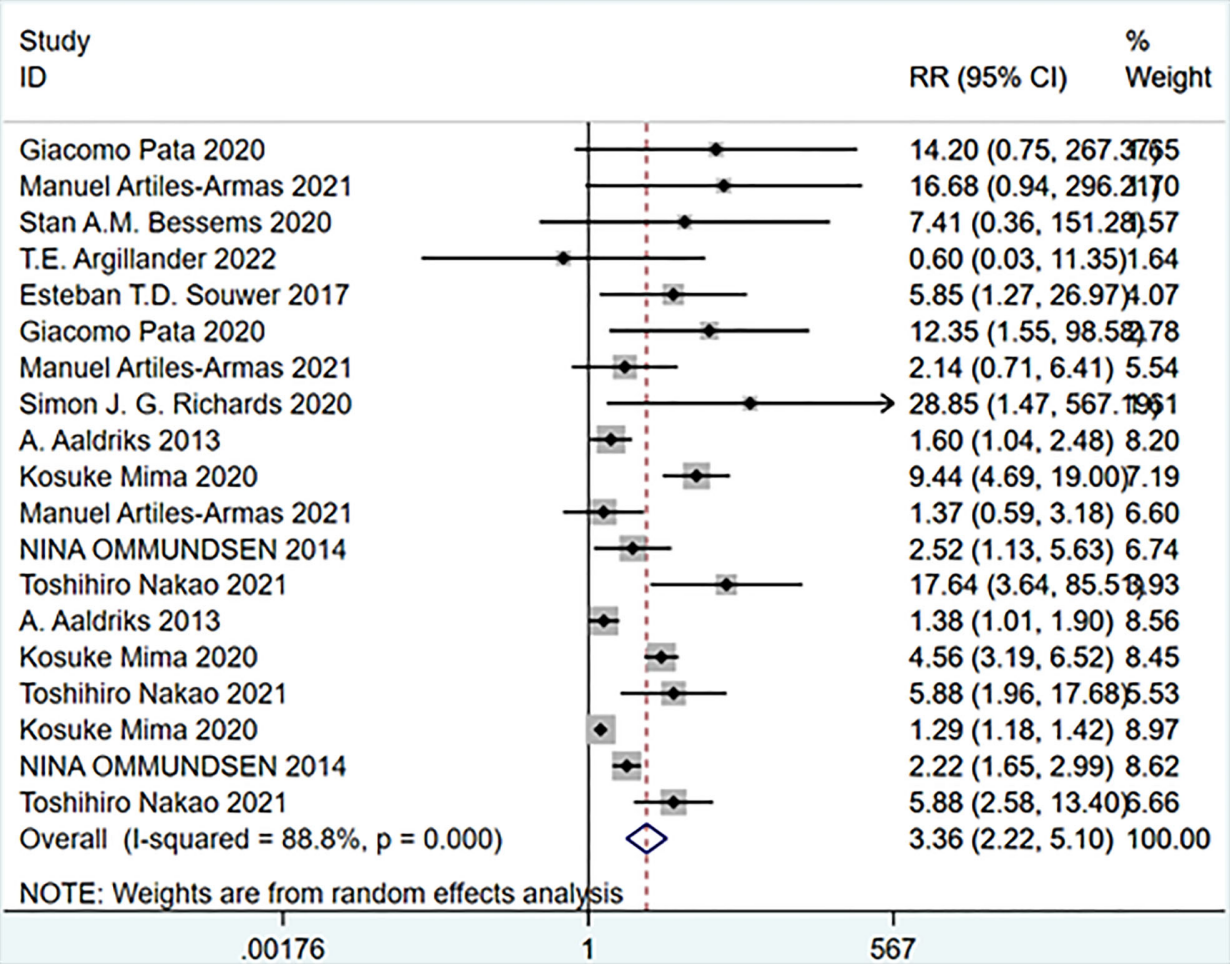
Mortality at different follow-up times.

A meta-analysis of studies grouped according to time to death after treatment showed that patients classified as frail had higher mortality rates than non-frail patients, either 30 days after treatment or 5 years after treatment. Both short- and long-term survival declines in colorectal cancer patients were associated with frailty. Heterogeneity between studies was observed in subgroup analyses (Overall: I-squared=88.8%, p=0.000, RR (95%CI) =3.36 (2.22, 5.10)) (**Figure 15**).

#### Frailty and complications of varying degrees after treatment

**Figure 16:**
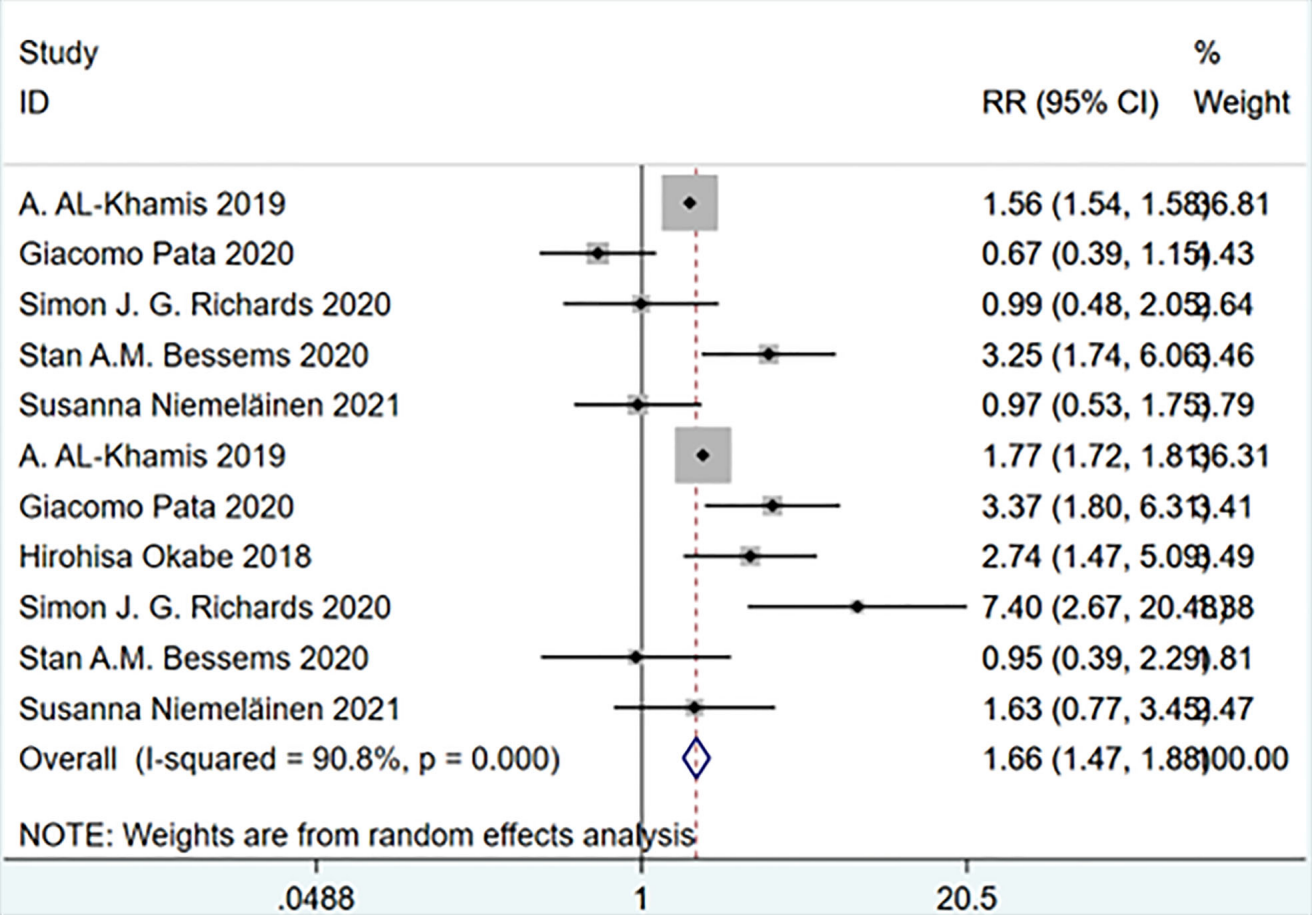
Complications of varying degrees after treatment.

A meta-analysis of grouped studies with minor or major complications after treatment showed that the incidence of minor complications was not statistically different between the frail and non-frail groups, suggesting that the likelihood of minor complications was similar in both groups; whereas the frail group was more likely to have serious complications than the non-frail group. Heterogeneity between studies was observed in the subgroup analysis. (Overall: I squared=90.8%, p=0.000, RR (95%CI) =1.66 (1.47, 1.88)) (**Figure 16**).

#### Publication bias and sensitivity analysis

**Figure 17:**
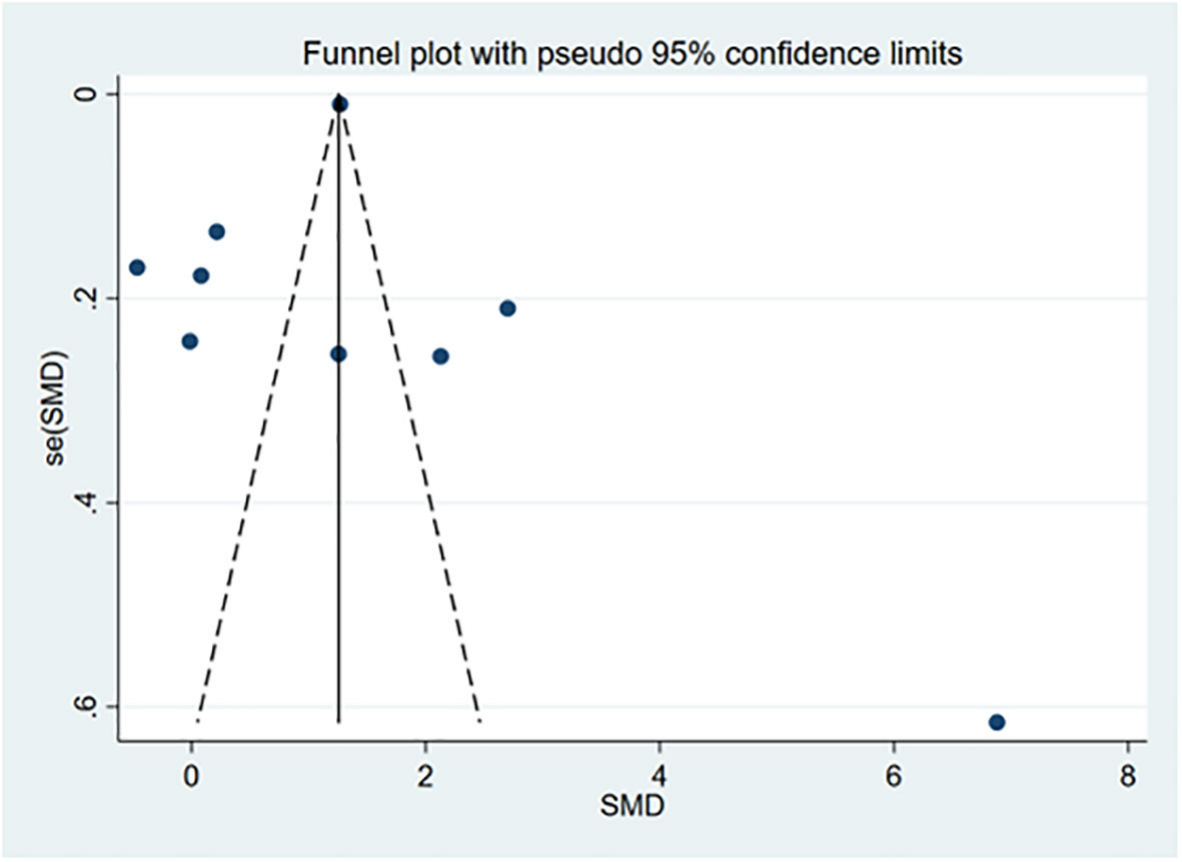
Funnel chart.

**Figure 18:**
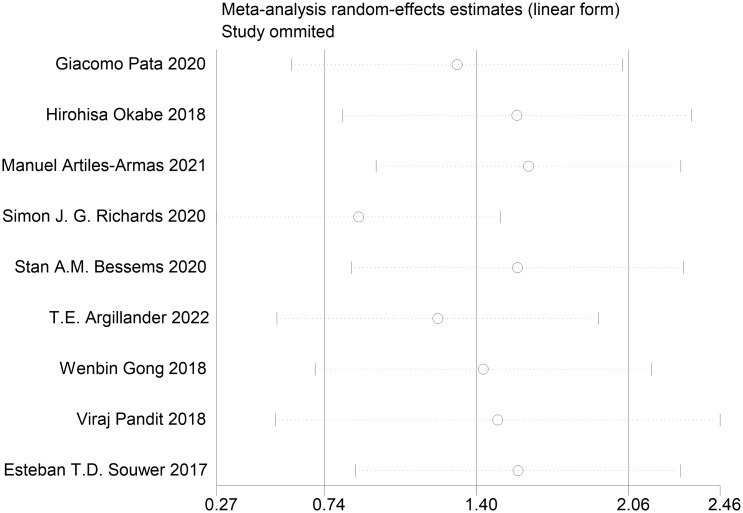
Sensitivity analysis.

**Figure 19:**
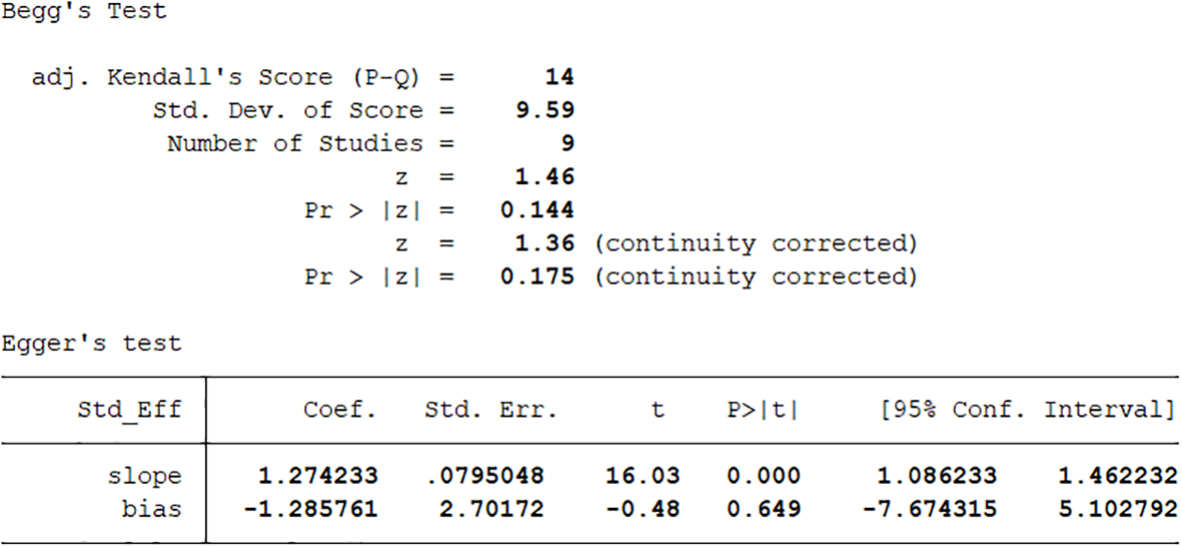
Begg & egger’s test.

We performed publication bias and sensitivity analyses for each outcome. Eliminating each study one by one did not change the direction of the effect size of any results, verifying that the results were stable (**Figures 17**–**19**).

## Discussion

Our meta-analysis showed that frail colorectal cancer patients had poor short-term or long-term outcomes. Short-term and long-term mortality and length of hospital stay were higher in frail patients than in non-frail patients. The odds of readmission, postoperative blood transfusion, insanity, and discharge destination other than home were also higher in frail patients than in non-frail patients. In terms of complications, there was no significant difference in the probability of minor complications between the two groups. In terms of serious complications, the frail group had a higher incidence than the non-frail group. We also found that frailty has an important impact on the prognosis of patients with colorectal cancer, regardless of frailty screening tools, reported from multiple studies (24–26).

In tests for heterogeneity, we found heterogeneity among some of the findings, especially in terms of hospital stay. Considering the interference of multiple factors such as study environment, study method, publication year, frailty screening methods, tumor location and stage, we speculate that the source of high heterogeneity may be very complex. Based on data from our research, we cannot complete the analysis of all sources of high heterogeneity. Although some studies included only colon cancer patients, some included emergency surgery in the analysis, and some had smaller sample sizes, these factors may not have had a significant effect on the overall results. It also did not show large errors in publication bias and sensitivity analysis. Overall, the studies had relatively reliable quality ratings.

It has been proved that the prevalence of frailty in older patients with colorectal cancer and an indication for surgery ranges from 25 to 46 percent, depending on the population studied and the tools used to measure it (27). Several frail screening tools have been shown to be useful in predicting surgical and chemotherapy outcomes (28), although not all validated tools have been studied. Studies have shown that the sensitivity, specificity, positive predictive value, and negative predictive value of predicting CGA depend on the tool used, the vulnerability in the sample, and the cutoff value chosen (29). Therefore, some limitations of existing fragile tools and existing fragile literature must be kept in mind when selecting fragile tools. The prevalence of frailty varies slightly from study to study depending on the frailty tool used; furthermore, the varying tools often do not identify exactly the same group of people (30, 31). Studies have also found that these scales differ in their ability to predict prognostic outcomes because different subgroups are analyzed (32). We hypothesized that the magnitude of the risk of death in frail colorectal cancer patients may depend on the type of frailty assessment scale used, and we had to examine these findings separately because there are so many frailty screening tools available. Screening for frailty has a variety of additional tools, including cognitive impairment, disability, and comorbidities. Thus there is still some debate as to which frailty screening tool is the yardstick. Even social and economic factors of frailty (e.g., poverty, social isolation) are raised. But whether these additional tools have the same validity as existing frailty tools requires more validation.

The underlying mechanisms between frailty and poor prognosis in colorectal cancer have not been extensively studied. Nonetheless, several studies have reported elevated levels of C-reactive protein, interleukin-6, and tumor necrosis factor alpha in frail patients, suggesting that chronic inflammation may play a role (33, 34). Thus, overt chronic inflammation in frail patients may compromise their immune system and further reduce their functional reserve to adapt to stress (34, 35). Therefore, they cannot tolerate the side effects of the treatment, resulting in incomplete treatment (36). Whether it’s surgery or chemotherapy, clinicians worry about whether patients, especially frail patients, will be able to tolerate the trauma and side effects of treatment (37–39). They may be more willing to reduce the risk, and the benefit of the treatment is also reduced. Frail patients may also have other geriatric syndromes and poor postoperative outcomes, which can also negatively impact their long-term prognosis. These factors may explain the worse prognosis observed in frail colorectal cancer patients.

In addition, factors affecting the prognosis of patients with colorectal cancer are not limited to frailty. Several studies have found that sarcopenia is also common in cancer patients and predicts longer hospital stay LOS, worse postoperative complications, susceptibility to chemotherapy toxicity, decreased quality of life, and poor survival (40–43). Studies suggest that inflammatory markers are related to sarcopenia and play a major role in the development of sarcopenia (44). When concentrations of inflammatory factors such as tumor necrosis factor and interleukin-6 are elevated, they activate multiple metabolic pathways, leading to reduced protein degradation and synthesis, and by disrupting insulin signaling, leading to insulin resistance, which further reduces muscle mass. Low-grade systemic inflammation caused by tumors may lead to local muscle inflammation, which in turn leads to muscle degeneration (45). Muscles are the basis for maintaining normal physiological activities of the human body. Therefore, under the influence of inflammation and sarcopenia, the prognosis of colorectal cancer patients is not optimistic.

Likewise, malnutrition affects outcomes in patients with colorectal cancer. Preoperative malnutrition in colorectal cancer patients is associated with many adverse postoperative outcomes and poorer prognosis. Malnourished patients have significant weight loss after surgery, are more likely to develop septic shock, and have increased requirements for postoperative blood transfusion, mechanical ventilation, and reoperation (46). Malnutrition may also lead to immunosuppression and, as a result, post-operative inflammation and infection problems are more frequent. In addition, micronutrient deficiencies may also lead to increased inflammation, lower serum albumin levels, and increased incidence of anastomotic leakage in patients (47). Patients with mild to severe malnutrition have significantly longer hospital stays and longer recovery of gastrointestinal function than well-nourished patients (48).

After patients are discharged from the hospital, they go to many different places. We consider that those patients who recover well will go back home to live with their families because they have retained some self-care ability. Those who have lost their independence mostly go to some nursing institutions or nursing homes, and they must live with the help of others. The latter were mostly those who were identified as frail.

We also found the results of postoperative blood transfusions and found that frail patients were more likely to require blood transfusions than non-frail patients. This may be due to the fact that most frail patients are already in a state of anemia, coupled with a weaker physiological reserve, which increases the difficulty of surgery and increases the risk of bleeding. After experiencing external stimuli such as surgery, it is more difficult for oneself to maintain a steady state, resulting in an external means-blood transfusion to help recovery.

Notably, not all adverse outcomes were associated with preoperative frailty (49). We found from the included studies that the stage of the tumor (TNM stage), the size and location of the tumor, the method of treatment (laparoscopy, laparotomy, radiotherapy, chemotherapy), whether there was intestinal obstruction before surgery, and whether there was a surgical stoma (temporary or permanent) may affect the prognosis of patients with colorectal cancer. Another study performed in oncological patients with different types of tumors and cancer stages found no relationship between preoperative frailty and postsurgical mortality (50), suggesting that, in the case of malignancies, factors other than frailty (tumor location and the presence of metastases) likely play a major role. This also confirms our conjecture.

Similarly, frail patients with advanced tumors and preoperative bowel obstruction or perforation tend to have worse outcomes. And those who are already frail can only receive palliative chemotherapy or local surgical resection. In this palliative treatment approach, frailty is often aggravated, leading to a vicious circle with a far worse prognosis than non-frail patients. In addition, after evaluation by MNA nutritional score, Barthel index, and ASA grading standard, patients under different grades also have different prognostic performance. But no research has yet confirmed their link to frailty, and whether they should be part of frailty screening.

Screening frailty as an independent risk stratification tool in colorectal cancer patients has become imperative. Standard treatment for able-bodied patients with colorectal cancer, while for frail patients with colorectal cancer, the need for an individualized treatment plan must be considered (51). Before treating patients, clinicians will use various evaluation tools to screen out frail patients so that they can receive more care and formulate more suitable programs.

A comprehensive frailty assessment of colorectal cancer patients not only facilitates the early identification and comprehensive management of frailty syndromes, but also can optimize clinical care by obtaining physical and psychological information about the patient. Future studies should evaluate the prognostic value of frailty in the diagnosis and treatment of colorectal cancer patients. Due to the increasing number of elderly CRC patients, their frailty is very common (52). However, in the current study, the lack of a unified screening tool for frailty and the incompleteness of the test results make the data for evaluating the prognosis of patients with colorectal cancer for frailty still lacking (53, 54). It is also worth noting that, the study found frailty may also interact with colorectal cancer, accelerating disease progression or worsening prognosis (55). Therefore, frailty may affect tumor biology, which may be an important line of thought for future research.

## Limitations

We searched extensively for eligible studies, but it is still possible that we missed some relevant studies in other languages or databases. In the heterogeneity test, only the length of hospital stay had a relatively large publication bias, but overall there was no obvious publication bias, and the results were relatively reliable. Also, the number of research articles we included and the sample size of the studies were not large enough. The mechanism between frailty and prognostic changes in patients with colorectal cancer is unclear. Therefore, more clinical data and mechanistic studies are needed to supplement.

## Conclusion

Frailty has a huge impact on the prognosis of colorectal cancer patients, especially in mortality and complications after treatment. To further explore how frailty alters the outcomes of colorectal cancer patients, and how to reduce the impact of this poor prognosis, more authoritative frailty assessment criteria and more clinical data are needed.

## Data availability statement

The original contributions presented in the study are included in the article/**Supplementary Material**. Further inquiries can be directed to the corresponding author.

## Author contributions

MC and YH conceived and designed this study; MC completed the literature search and screening; JL and YJ included and excluded the literature and completed the quality assessment; ZG completed the data extraction; MC and ZG completed statistical analysis; MC completed the manuscript. All authors contributed to the article and approved the submitted version.

## Acknowledgments

The authors acknowledge Frontiers in Oncology team. They also particularly thank the reviewers and editors for their valuable comments, which helped considerably to improve the quality of the manuscript.

## Conflict of interest

The author declares that the research was conducted in the absence of any commercial or financial relationships that could be construed as a potential conflict of interest.

## Publisher’s note

All claims expressed in this article are solely those of the authors and do not necessarily represent those of their affiliated organizations, or those of the publisher, the editors and the reviewers. Any product that may be evaluated in this article, or claim that may be made by its manufacturer, is not guaranteed or endorsed by the publisher.
