# Supplementary material for: Frailty affects prognosis in patients with colorectal cancer: A systematic review and meta-analysis
Source: Front Oncol. 2022 Nov 3;12:1017183. doi: 10.3389/fonc.2022.1017183 (PMC9669723; doi:10.3389/fonc.2022.1017183)
Supplement: Supplementary file 1 [file DataSheet_1.zip › Appendix A.DOCX]

PubMed

("Frailty"[MeSH Terms] OR "Frailties"[Title/Abstract] OR "Frailness"[Title/Abstract] OR "frailty syndrome"[Title/Abstract] OR "Debility"[Title/Abstract] OR "Debilities"[Title/Abstract]) AND 2012/01/01:2022/12/31[Date - Publication] AND (("colorectal neoplasms"[MeSH Terms] OR "colorectal neoplasm"[Title/Abstract] OR "neoplasm colorectal"[Title/Abstract] OR "neoplasms colorectal"[Title/Abstract] OR "colorectal tumors"[Title/Abstract] OR "colorectal tumor"[Title/Abstract] OR "tumor colorectal"[Title/Abstract] OR "tumors colorectal"[Title/Abstract] OR "colorectal cancer"[Title/Abstract] OR "cancer colorectal"[Title/Abstract] OR "cancers colorectal"[Title/Abstract] OR "colorectal cancers"[Title/Abstract] OR "colorectal carcinoma"[Title/Abstract] OR "carcinoma colorectal"[Title/Abstract] OR "carcinomas colorectal"[Title/Abstract] OR "colorectal carcinomas"[Title/Abstract]) AND 2012/01/01:2022/12/31[Date - Publication])

("Frailty"[MeSH Terms] OR "Frailties"[Title/Abstract] OR "Frailness"[Title/Abstract] OR "frailty syndrome"[Title/Abstract] OR "Debility"[Title/Abstract] OR "Debilities"[Title/Abstract]) AND 2012/01/01:2022/12/31[Date - Publication] AND (("Neoplasms"[MeSH Terms] OR "Neoplasm"[Title/Abstract] OR "Tumor"[Title/Abstract] OR "Tumors"[Title/Abstract] OR "Neoplasia"[Title/Abstract] OR "Neoplasias"[Title/Abstract] OR "Cancer"[Title/Abstract] OR "Cancers"[Title/Abstract] OR "malignant neoplasm"[Title/Abstract] OR "Malignancy"[Title/Abstract] OR "Malignancies"[Title/Abstract] OR "malignant neoplasms"[Title/Abstract] OR "neoplasm malignant"[Title/Abstract] OR "neoplasms malignant"[Title/Abstract] OR "benign neoplasms"[Title/Abstract] OR "benign neoplasm"[Title/Abstract] OR "neoplasms benign"[Title/Abstract] OR "neoplasm benign"[Title/Abstract]) AND 2012/01/01:2022/12/31[Date - Publication] AND (("rectum"[MeSH Terms] AND 2012/01/01:2022/12/31[Date - Publication]) OR (("colon"[MeSH Terms] OR "taenia coli"[Title/Abstract] OR "appendix epiploica"[Title/Abstract] OR "omental appendix"[Title/Abstract] OR (("Appendix"[MeSH Terms] OR "Appendix"[All Fields] OR "appendix s"[All Fields] OR "appendixes"[All Fields]) AND "Omental"[Title/Abstract]) OR "omental appendices"[Title/Abstract] OR (("appendice"[All Fields] OR "appendiceal"[All Fields] OR "Appendices"[All Fields]) AND "Omental"[Title/Abstract])) AND 2012/01/01:2022/12/31[Date - Publication])))

**Appendix A: Search strategy on PubMed**

| #1 | (((((Frailty[MeSH Terms]) OR (Frailties[Title/Abstract])) OR (Frailness[Title/Abstract])) OR (Frailty Syndrome[Title/Abstract])) OR (Debility[Title/Abstract])) OR (Debilities[Title/Abstract]) |
| --- | --- |
| #2 | (((((((((((((((Colorectal Neoplasms[MeSH Terms]) OR (Colorectal Neoplasm[Title/Abstract])) OR (Neoplasm, Colorectal[Title/Abstract])) OR (Neoplasms, Colorectal[Title/Abstract])) OR (Colorectal Tumors[Title/Abstract])) OR (Colorectal Tumor[Title/Abstract])) OR (Tumor, Colorectal[Title/Abstract])) OR (Tumors, Colorectal[Title/Abstract])) OR (Colorectal Cancer[Title/Abstract])) OR (Cancer, Colorectal[Title/Abstract])) OR (Cancers, Colorectal[Title/Abstract])) OR (Colorectal Cancers[Title/Abstract])) OR (Colorectal Carcinoma[Title/Abstract])) OR (Carcinoma, Colorectal[Title/Abstract])) OR (Carcinomas, Colorectal[Title/Abstract])) OR (Colorectal Carcinomas[Title/Abstract]) |
| #3 | #1 AND #2 |
| #4 | (((((((((((((((((Neoplasms[MeSH Terms]) OR (Neoplasm[Title/Abstract])) OR (Tumor[Title/Abstract])) OR (Tumors[Title/Abstract])) OR (Neoplasia[Title/Abstract])) OR (Neoplasias[Title/Abstract])) OR (Cancer[Title/Abstract])) OR (Cancers[Title/Abstract])) OR (Malignant Neoplasm[Title/Abstract])) OR (Malignancy[Title/Abstract])) OR (Malignancies[Title/Abstract])) OR (Malignant Neoplasms[Title/Abstract])) OR (Neoplasm, Malignant[Title/Abstract])) OR (Neoplasms, Malignant[Title/Abstract])) OR (Benign Neoplasms[Title/Abstract])) OR (Benign Neoplasm[Title/Abstract])) OR (Neoplasms, Benign[Title/Abstract])) OR (Neoplasm, Benign[Title/Abstract]) |
| #5 | Rectum[MeSH Terms] |
| #6 | ((((((Colon[MeSH Terms]) OR (Taenia Coli[Title/Abstract])) OR (Appendix Epiploica[Title/Abstract])) OR (Omental Appendix[Title/Abstract])) OR (Appendix, Omental[Title/Abstract])) OR (Omental Appendices[Title/Abstract])) OR (Appendices, Omental[Title/Abstract]) |
| #7 | (#5 OR #6) AND #4 |
| #8 | #1 AND #7 |
| #9 | #3 OR #8 |
