# Supplementary material for: Frailty affects prognosis in patients with colorectal cancer: A systematic review and meta-analysis
Source: Front Oncol. 2022 Nov 3;12:1017183. doi: 10.3389/fonc.2022.1017183 (PMC9669723; doi:10.3389/fonc.2022.1017183)
Supplement: Supplementary file 1 [file DataSheet_1.zip › Appendix B.DOCX]

**Appendix B: Search strategy on PubMed**

| #1 | "frailty"[MeSH Terms] OR "frailty"[All Fields] OR "frailties"[All Fields] OR ("frailty"[MeSH Terms] OR "frailty"[All Fields] OR "frailties"[All Fields]) OR ("frail"[All Fields] OR "frails"[All Fields] OR "frailty"[MeSH Terms] OR "frailty"[All Fields] OR "frailness"[All Fields]) OR ("frailty"[MeSH Terms] OR "frailty"[All Fields] OR ("frailty"[All Fields] AND "syndrome"[All Fields]) OR "frailty syndrome"[All Fields]) OR ("frailty"[MeSH Terms] OR "frailty"[All Fields] OR "debility"[All Fields]) OR ("frailty"[MeSH Terms] OR "frailty"[All Fields] OR "debilities"[All Fields]) |
| --- | --- |
| #2 | "colorectal neoplasms"[MeSH Terms] OR ("colorectal"[All Fields] AND "neoplasms"[All Fields]) OR "colorectal neoplasms"[All Fields] OR ("colorectal neoplasms"[MeSH Terms] OR ("colorectal"[All Fields] AND "neoplasms"[All Fields]) OR "colorectal neoplasms"[All Fields] OR ("colorectal"[All Fields] AND "neoplasm"[All Fields]) OR "colorectal neoplasm"[All Fields]) OR ("colorectal neoplasms"[MeSH Terms] OR ("colorectal"[All Fields] AND "neoplasms"[All Fields]) OR "colorectal neoplasms"[All Fields] OR ("neoplasm"[All Fields] AND "colorectal"[All Fields]) OR "neoplasm colorectal"[All Fields]) OR ("colorectal neoplasms"[MeSH Terms] OR ("colorectal"[All Fields] AND "neoplasms"[All Fields]) OR "colorectal neoplasms"[All Fields] OR ("neoplasms"[All Fields] AND "colorectal"[All Fields]) OR "neoplasms colorectal"[All Fields]) OR ("colorectal tumours"[All Fields] OR "colorectal neoplasms"[MeSH Terms] OR ("colorectal"[All Fields] AND "neoplasms"[All Fields]) OR "colorectal neoplasms"[All Fields] OR ("colorectal"[All Fields] AND "tumors"[All Fields]) OR "colorectal tumors"[All Fields]) OR ("colorectal tumour"[All Fields] OR "colorectal neoplasms"[MeSH Terms] OR ("colorectal"[All Fields] AND "neoplasms"[All Fields]) OR "colorectal neoplasms"[All Fields] OR ("colorectal"[All Fields] AND "tumor"[All Fields]) OR "colorectal tumor"[All Fields]) OR ("colorectal neoplasms"[MeSH Terms] OR ("colorectal"[All Fields] AND "neoplasms"[All Fields]) OR "colorectal neoplasms"[All Fields] OR ("tumor"[All Fields] AND "colorectal"[All Fields]) OR "tumor colorectal"[All Fields]) OR ("colorectal neoplasms"[MeSH Terms] OR ("colorectal"[All Fields] AND "neoplasms"[All Fields]) OR "colorectal neoplasms"[All Fields] OR ("tumors"[All Fields] AND "colorectal"[All Fields]) OR "tumors colorectal"[All Fields]) OR ("colorectal neoplasms"[MeSH Terms] OR ("colorectal"[All Fields] AND "neoplasms"[All Fields]) OR "colorectal neoplasms"[All Fields] OR ("colorectal"[All Fields] AND "cancer"[All Fields]) OR "colorectal cancer"[All Fields]) OR ("colorectal neoplasms"[MeSH Terms] OR ("colorectal"[All Fields] AND "neoplasms"[All Fields]) OR "colorectal neoplasms"[All Fields] OR ("cancer"[All Fields] AND "colorectal"[All Fields]) OR "cancer colorectal"[All Fields]) OR ("colorectal neoplasms"[MeSH Terms] OR ("colorectal"[All Fields] AND "neoplasms"[All Fields]) OR "colorectal neoplasms"[All Fields] OR ("cancers"[All Fields] AND "colorectal"[All Fields]) OR "cancers colorectal"[All Fields]) OR ("colorectal neoplasms"[MeSH Terms] OR ("colorectal"[All Fields] AND "neoplasms"[All Fields]) OR "colorectal neoplasms"[All Fields] OR ("colorectal"[All Fields] AND "cancers"[All Fields]) OR "colorectal cancers"[All Fields]) OR ("colorectal neoplasms"[MeSH Terms] OR ("colorectal"[All Fields] AND "neoplasms"[All Fields]) OR "colorectal neoplasms"[All Fields] OR ("colorectal"[All Fields] AND "carcinoma"[All Fields]) OR "colorectal carcinoma"[All Fields]) OR ("colorectal neoplasms"[MeSH Terms] OR ("colorectal"[All Fields] AND "neoplasms"[All Fields]) OR "colorectal neoplasms"[All Fields] OR ("carcinoma"[All Fields] AND "colorectal"[All Fields]) OR "carcinoma colorectal"[All Fields]) OR ("colorectal neoplasms"[MeSH Terms] OR ("colorectal"[All Fields] AND "neoplasms"[All Fields]) OR "colorectal neoplasms"[All Fields] OR ("carcinomas"[All Fields] AND "colorectal"[All Fields]) OR "carcinomas colorectal"[All Fields]) OR ("colorectal neoplasms"[MeSH Terms] OR ("colorectal"[All Fields] AND "neoplasms"[All Fields]) OR "colorectal neoplasms"[All Fields] OR ("colorectal"[All Fields] AND "carcinomas"[All Fields]) OR "colorectal carcinomas"[All Fields]) |
| #3 | #1 AND #2（490） |
| #4 | "neoplasm s"[All Fields] OR "neoplasms"[MeSH Terms] OR "neoplasms"[All Fields] OR "neoplasm"[All Fields] OR ("neoplasm s"[All Fields] OR "neoplasms"[MeSH Terms] OR "neoplasms"[All Fields] OR "neoplasm"[All Fields]) OR ("cysts"[MeSH Terms] OR "cysts"[All Fields] OR "cyst"[All Fields] OR "neurofibroma"[MeSH Terms] OR "neurofibroma"[All Fields] OR "neurofibromas"[All Fields] OR "tumor s"[All Fields] OR "tumoral"[All Fields] OR "tumorous"[All Fields] OR "tumour"[All Fields] OR "neoplasms"[MeSH Terms] OR "neoplasms"[All Fields] OR "tumor"[All Fields] OR "tumour s"[All Fields] OR "tumoural"[All Fields] OR "tumourous"[All Fields] OR "tumours"[All Fields] OR "tumors"[All Fields]) OR ("cysts"[MeSH Terms] OR "cysts"[All Fields] OR "cyst"[All Fields] OR "neurofibroma"[MeSH Terms] OR "neurofibroma"[All Fields] OR "neurofibromas"[All Fields] OR "tumor s"[All Fields] OR "tumoral"[All Fields] OR "tumorous"[All Fields] OR "tumour"[All Fields] OR "neoplasms"[MeSH Terms] OR "neoplasms"[All Fields] OR "tumor"[All Fields] OR "tumour s"[All Fields] OR "tumoural"[All Fields] OR "tumourous"[All Fields] OR "tumours"[All Fields] OR "tumors"[All Fields]) OR ("neoplasms"[MeSH Terms] OR "neoplasms"[All Fields] OR "neoplasia"[All Fields] OR "neoplasias"[All Fields]) OR ("neoplasms"[MeSH Terms] OR "neoplasms"[All Fields] OR "neoplasia"[All Fields] OR "neoplasias"[All Fields]) OR ("cancer s"[All Fields] OR "cancerated"[All Fields] OR "canceration"[All Fields] OR "cancerization"[All Fields] OR "cancerized"[All Fields] OR "cancerous"[All Fields] OR "neoplasms"[MeSH Terms] OR "neoplasms"[All Fields] OR "cancer"[All Fields] OR "cancers"[All Fields]) OR ("cancer s"[All Fields] OR "cancerated"[All Fields] OR "canceration"[All Fields] OR "cancerization"[All Fields] OR "cancerized"[All Fields] OR "cancerous"[All Fields] OR "neoplasms"[MeSH Terms] OR "neoplasms"[All Fields] OR "cancer"[All Fields] OR "cancers"[All Fields]) OR ("neoplasms"[MeSH Terms] OR "neoplasms"[All Fields] OR ("malignant"[All Fields] AND "neoplasm"[All Fields]) OR "malignant neoplasm"[All Fields]) OR ("malign"[All Fields] OR "malignance"[All Fields] OR "malignances"[All Fields] OR "malignant"[All Fields] OR "malignants"[All Fields] OR "malignities"[All Fields] OR "malignity"[All Fields] OR "malignization"[All Fields] OR "malignized"[All Fields] OR "maligns"[All Fields] OR "neoplasms"[MeSH Terms] OR "neoplasms"[All Fields] OR "malignancies"[All Fields] OR "malignancy"[All Fields]) OR ("malign"[All Fields] OR "malignance"[All Fields] OR "malignances"[All Fields] OR "malignant"[All Fields] OR "malignants"[All Fields] OR "malignities"[All Fields] OR "malignity"[All Fields] OR "malignization"[All Fields] OR "malignized"[All Fields] OR "maligns"[All Fields] OR "neoplasms"[MeSH Terms] OR "neoplasms"[All Fields] OR "malignancies"[All Fields] OR "malignancy"[All Fields]) OR ("neoplasms"[MeSH Terms] OR "neoplasms"[All Fields] OR ("malignant"[All Fields] AND "neoplasms"[All Fields]) OR "malignant neoplasms"[All Fields]) OR ("neoplasms"[MeSH Terms] OR "neoplasms"[All Fields] OR ("neoplasm"[All Fields] AND "malignant"[All Fields]) OR "neoplasm malignant"[All Fields]) OR ("neoplasms"[MeSH Terms] OR "neoplasms"[All Fields] OR ("neoplasms"[All Fields] AND "malignant"[All Fields]) OR "neoplasms malignant"[All Fields]) OR ("neoplasms"[MeSH Terms] OR "neoplasms"[All Fields] OR ("benign"[All Fields] AND "neoplasms"[All Fields]) OR "benign neoplasms"[All Fields]) OR ("neoplasms"[MeSH Terms] OR "neoplasms"[All Fields] OR ("benign"[All Fields] AND "neoplasm"[All Fields]) OR "benign neoplasm"[All Fields]) OR ("neoplasms"[MeSH Terms] OR "neoplasms"[All Fields] OR ("neoplasms"[All Fields] AND "benign"[All Fields]) OR "neoplasms benign"[All Fields]) OR ("neoplasms"[MeSH Terms] OR "neoplasms"[All Fields] OR ("neoplasm"[All Fields] AND "benign"[All Fields]) OR "neoplasm benign"[All Fields]) |
| #5 | "rectum"[MeSH Terms] OR "rectum"[All Fields] OR "rectums"[All Fields] |
| #6 | "colon"[MeSH Terms] OR "colon"[All Fields] OR "colonic"[All Fields] OR "colons"[All Fields] OR "colon s"[All Fields] OR "colonal"[All Fields] OR "colonically"[All Fields] OR "colonitis"[All Fields] OR ("colon"[MeSH Terms] OR "colon"[All Fields] OR ("taenia"[All Fields] AND "coli"[All Fields]) OR "taenia coli"[All Fields]) OR ("colon"[MeSH Terms] OR "colon"[All Fields] OR ("appendix"[All Fields] AND "epiploica"[All Fields]) OR "appendix epiploica"[All Fields]) OR ("colon"[MeSH Terms] OR "colon"[All Fields] OR ("omental"[All Fields] AND "appendix"[All Fields]) OR "omental appendix"[All Fields]) OR ("colon"[MeSH Terms] OR "colon"[All Fields] OR ("appendix"[All Fields] AND "omental"[All Fields])) OR ("colon"[MeSH Terms] OR "colon"[All Fields] OR ("omental"[All Fields] AND "appendices"[All Fields]) OR "omental appendices"[All Fields]) OR ("colon"[MeSH Terms] OR "colon"[All Fields] OR ("appendices"[All Fields] AND "omental"[All Fields])) |
| #7 | (#5 OR #6) AND #4 |
| #8 | #1 AND #7（211） |
| #9 | #3 OR #8（701） |

**Search strategy on Web of Science**

# Web of Science retrieval strategy (v0.1)

# databases: all databases

# retrieve:

16: TS=(Colorectal Neoplasms OR Colorectal Neoplasm OR Neoplasm, Colorectal OR Neoplasms, Colorectal OR Colorectal Tumors OR Colorectal Tumor OR Tumor, Colorectal OR Tumors, Colorectal OR Colorectal Cancer OR Cancer, Colorectal OR Cancers, Colorectal OR Colorectal Cancers OR Colorectal Carcinoma OR Carcinoma, Colorectal OR Carcinomas, Colorectal OR Colorectal Carcinomas) Search Results: 354455

17: TS=(Frailty OR Frailties OR Frailties OR Frailty Syndrome OR Debility OR Debilities) Search Results: 40259

18: TS=(Colon OR Taenia Coli OR Appendix Epiploica OR Omental Appendix OR Appendix, Omental OR Omental Appendices OR Appendices, Omental) Search Results: 476086

19: TS=(Rectum) Search Results: 128884

20: TS=(Neoplasms OR Neoplasm OR Tumor OR Tumors OR Neoplasia OR Neoplasias OR Cancer OR Cancers OR Malignant Neoplasm OR Malignancy OR Malignancies OR Malignant Neoplasms OR Neoplasm, Malignant OR Neoplasms, Malignant OR Benign Neoplasms OR Benign Neoplasm OR Neoplasms, Benign OR Neoplasm, Benign) Search Results: 8665903

21: #16 AND #17 Search Results: 515

22: #18 OR #19 Search Results: 557002

23: #22 AND #20 Search Results: 337376

24: #23 AND #17 Search Results: 189

**Search strategy on Embase**

Embase

Session Results

.......................................................

No. Query Results Results Date

#9. #1 AND #8 332 15 Sep 2022

#8. #6 AND #7 343,448 15 Sep 2022

#7. neoplasms OR neoplasm OR tumor OR tumors OR 6,687,523 15 Sep 2022

neoplasia OR neoplasias OR cancer OR cancers OR

(malignant AND neoplasm) OR malignancy OR

malignancies OR (malignant AND neoplasms) OR

(neoplasm, AND malignant) OR (neoplasms, AND

malignant) OR (benign AND neoplasms) OR (benign

AND neoplasm) OR (neoplasms, AND benign) OR

(neoplasm, AND benign)

#6. #4 OR #5 578,275 15 Sep 2022

#5. rectum 205,134 15 Sep 2022

#4. colon OR (taenia AND coli) OR (appendix AND 453,297 15 Sep 2022

epiploica) OR (omental AND appendix) OR

(appendix, AND omental) OR (omental AND

appendices) OR (appendices, AND omental)

#3. #1 AND #2 636 15 Sep 2022

#2. colorectal AND neoplasms OR (colorectal AND 321,366 15 Sep 2022

neoplasm) OR (neoplasm, AND colorectal) OR

(neoplasms, AND colorectal) OR (colorectal AND

tumors) OR (colorectal AND tumor) OR (tumor, AND

colorectal) OR (tumors, AND colorectal) OR

(colorectal AND cancer) OR (cancer, AND

colorectal) OR (cancers, AND colorectal) OR

(colorectal AND cancers) OR (colorectal AND

carcinoma) OR (carcinoma, AND colorectal) OR

(carcinomas, AND colorectal) OR (colorectal AND

carcinomas)

#1. frailty OR frailties OR frailness OR (frailty AND 39,315 15 Sep 2022

syndrome) OR debility OR debilities
