# Supplementary material for: Frailty affects prognosis in patients with colorectal cancer: A systematic review and meta-analysis
Source: Front Oncol. 2022 Nov 3;12:1017183. doi: 10.3389/fonc.2022.1017183 (PMC9669723; doi:10.3389/fonc.2022.1017183)
Supplement: Supplementary file 1 [file DataSheet_1.zip › Supplementary Figures.pdf]

## *Supplementary Figures*

Figure 1. Flow diagram of the selection strategy.

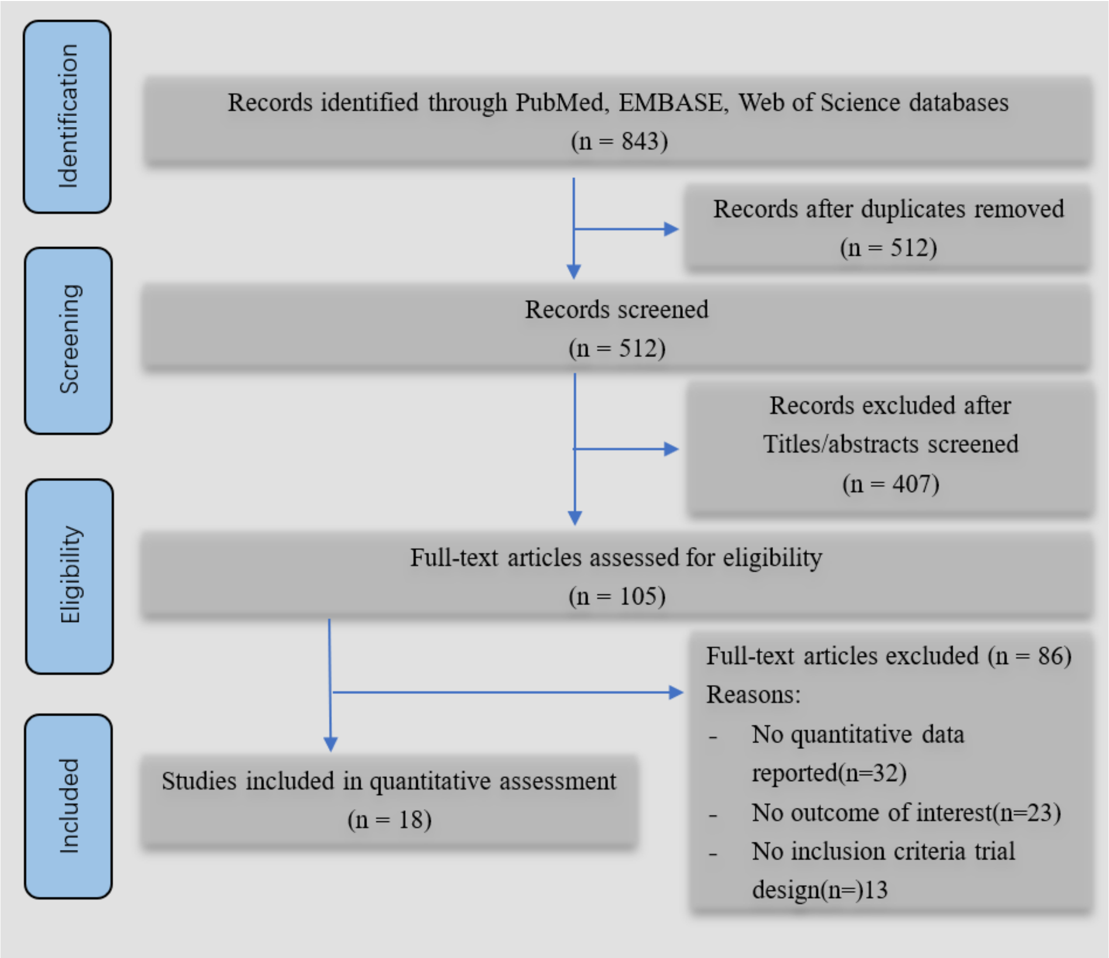

Figure 2: 30-day mortality

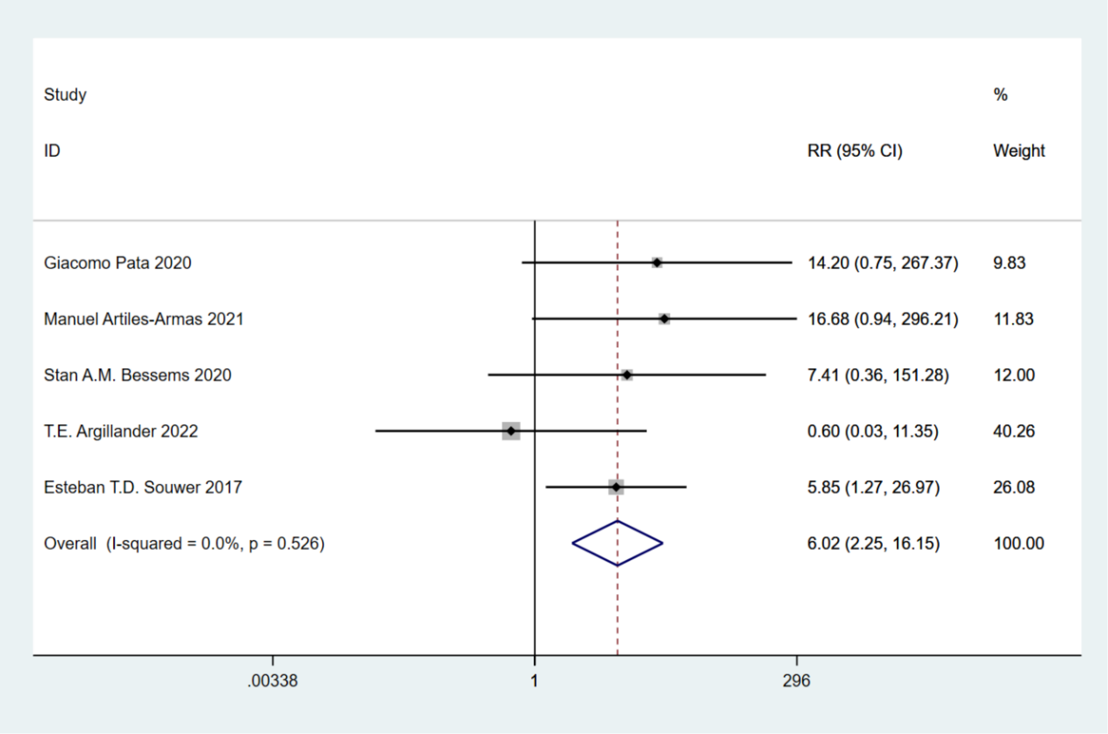


Figure 3: 90-day mortality

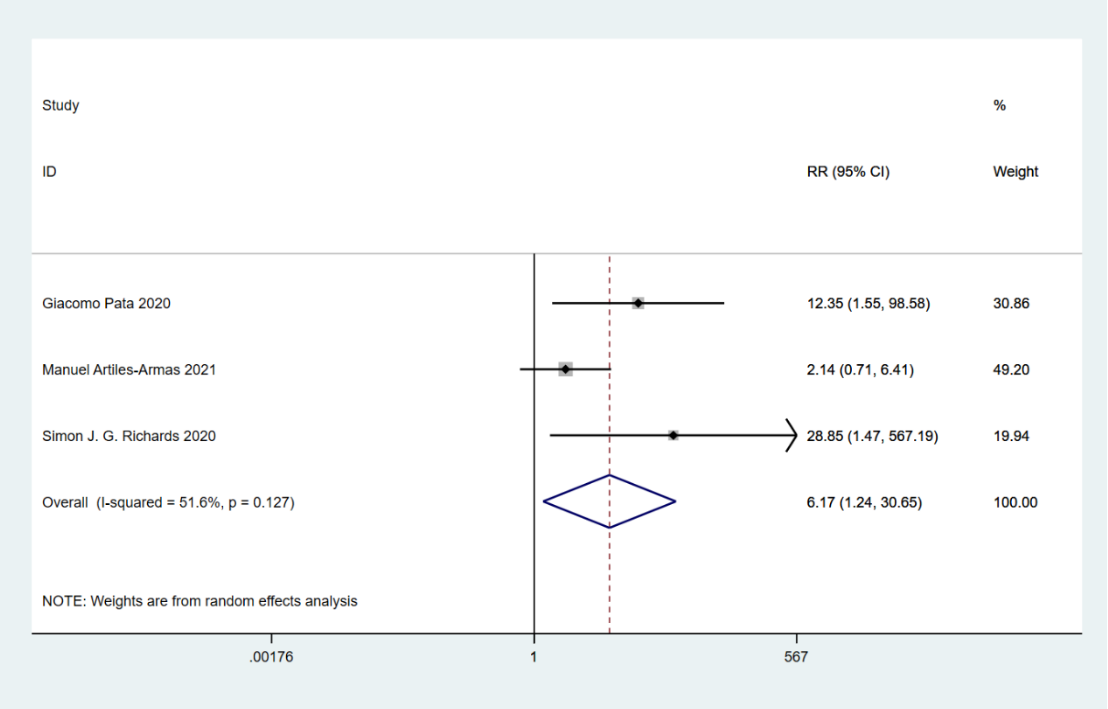

Figure 4: 1-year mortality

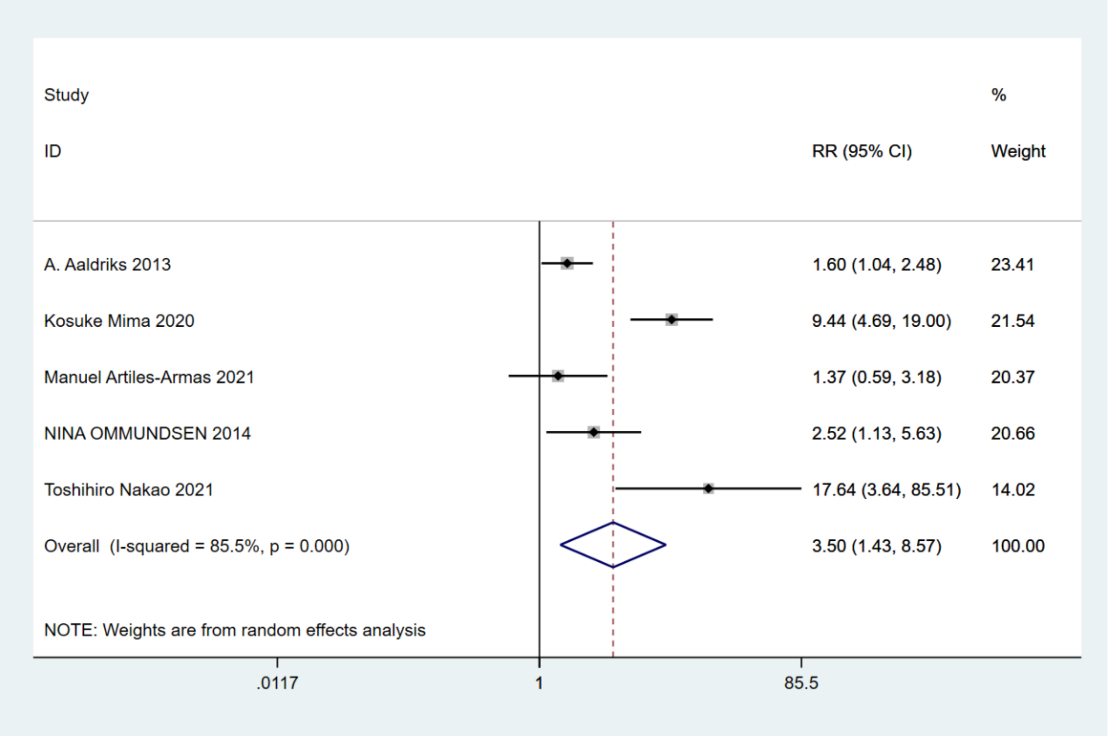


Figure 5: 2-year mortality

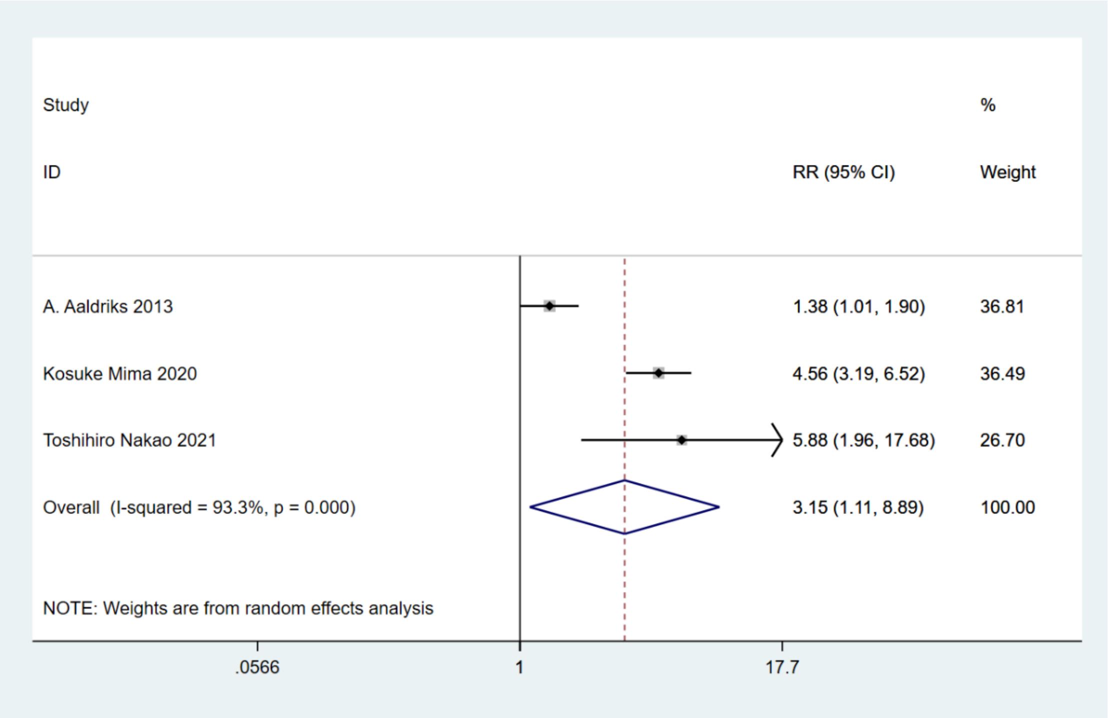

Figure 6: 5-year mortality

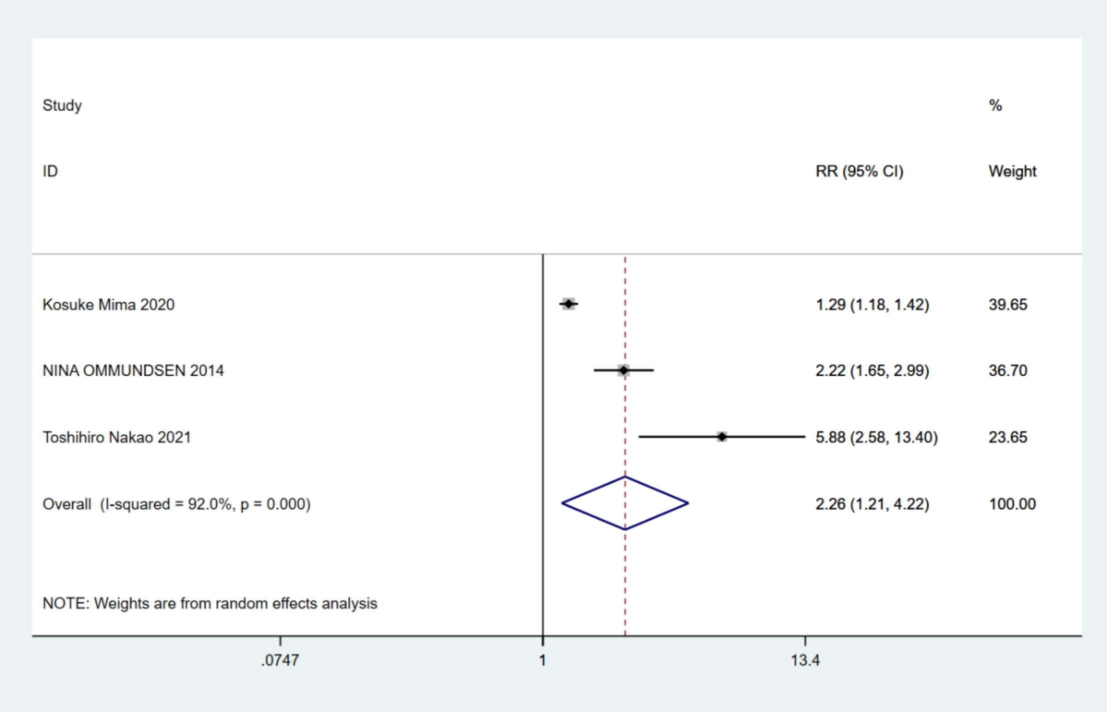


（Clavien–Dindo grade）

Figure 7: total complications

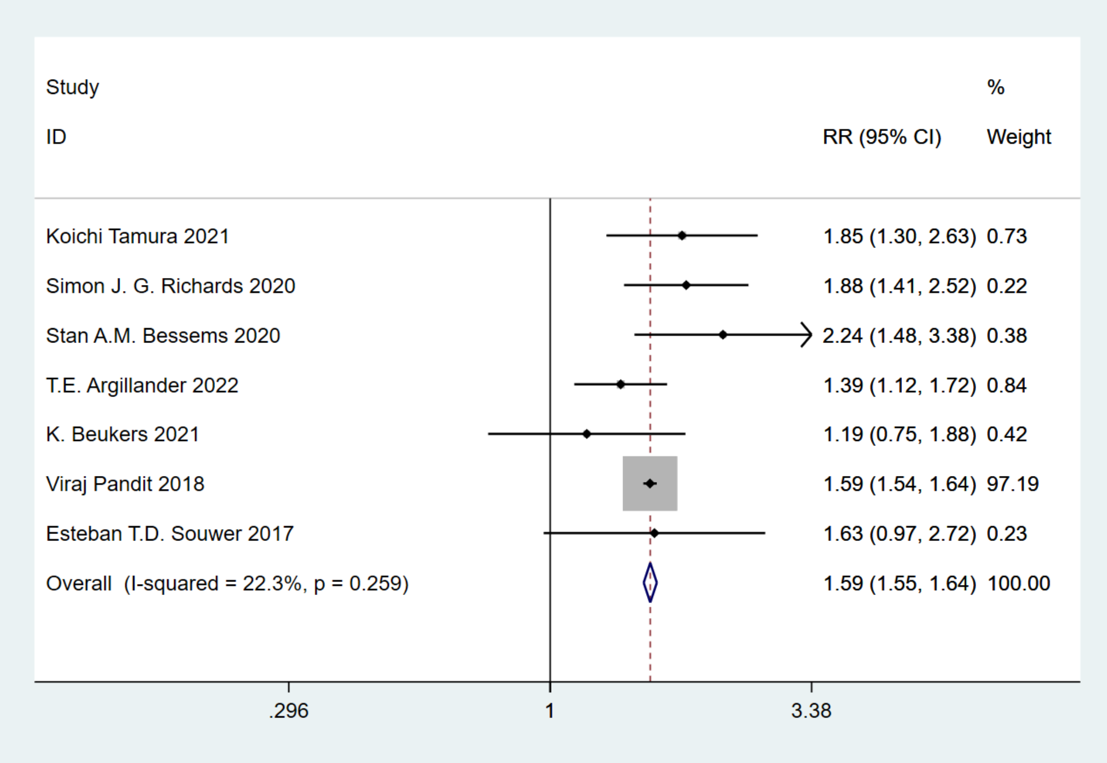

Figure 8: minor complications

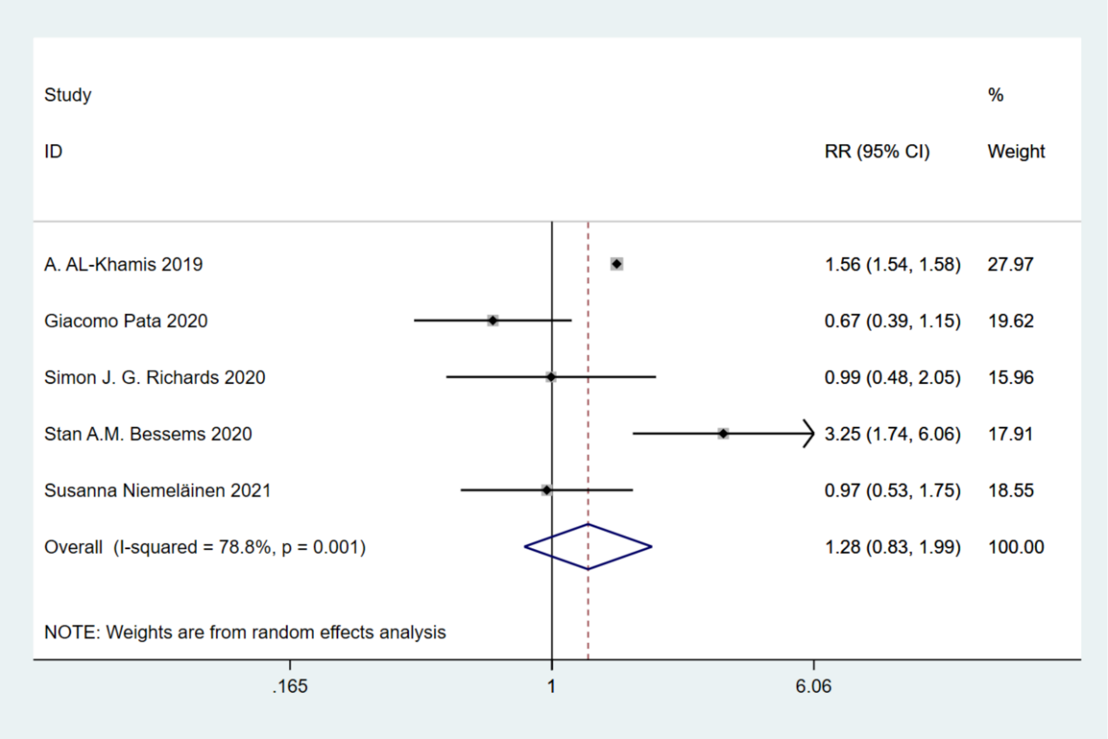


Figure 9: severe complications

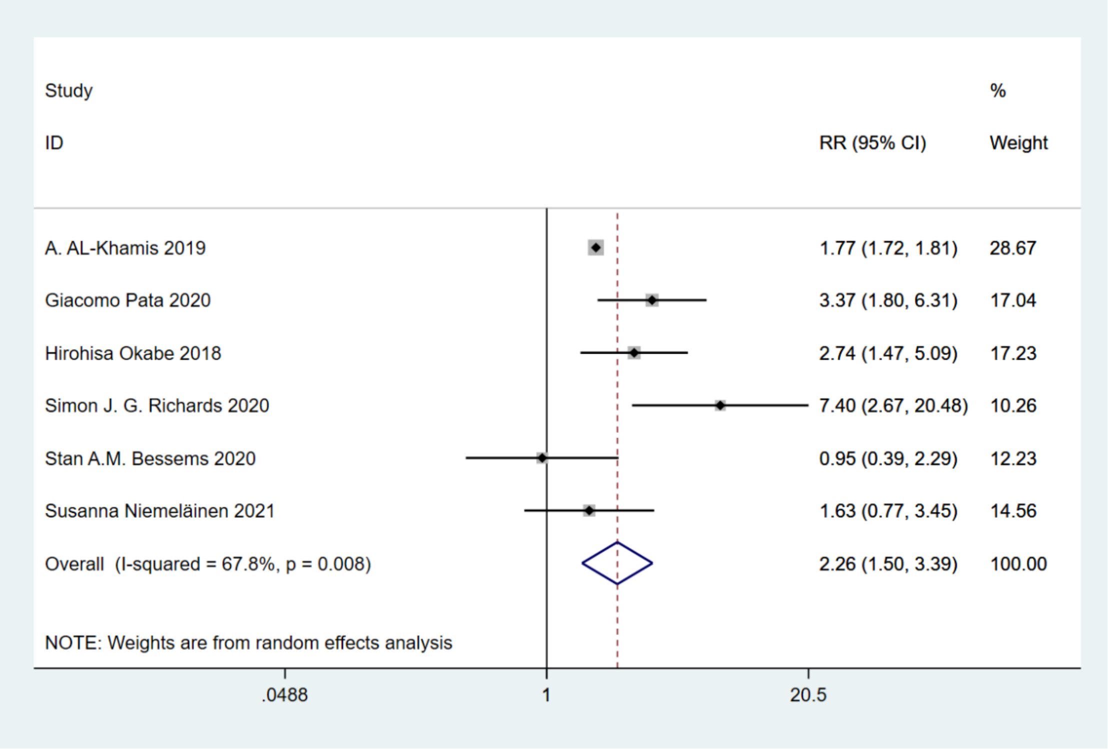

Figure 10: Delirium

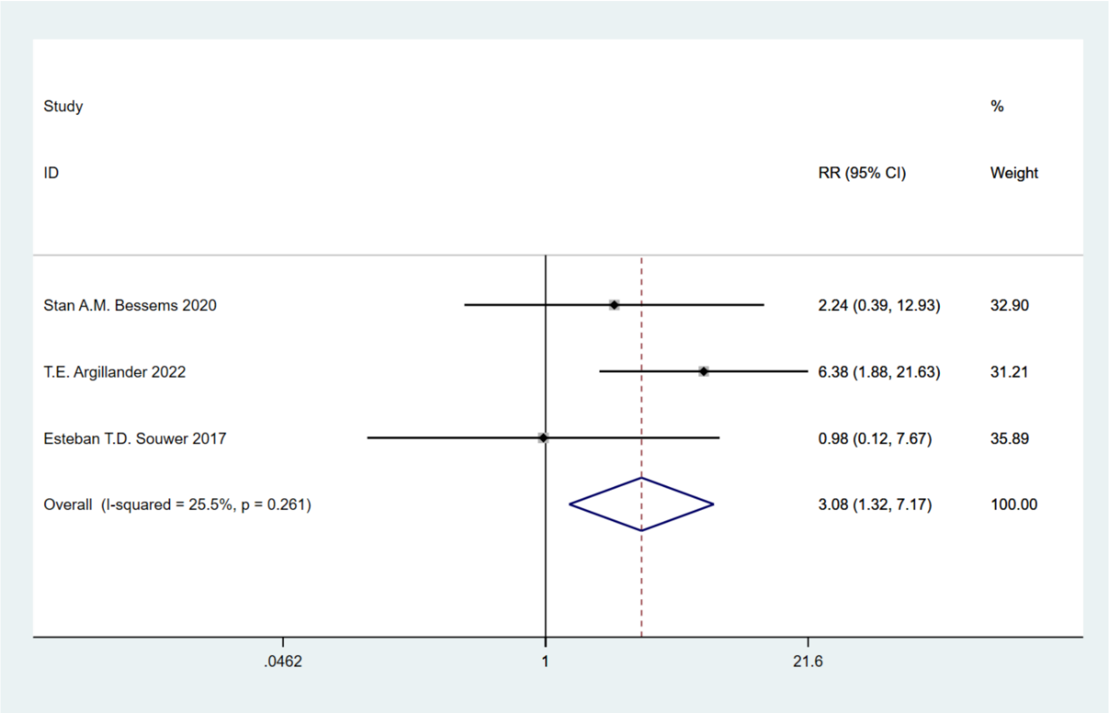


Figure 11: Postoperative Blood Transfusion

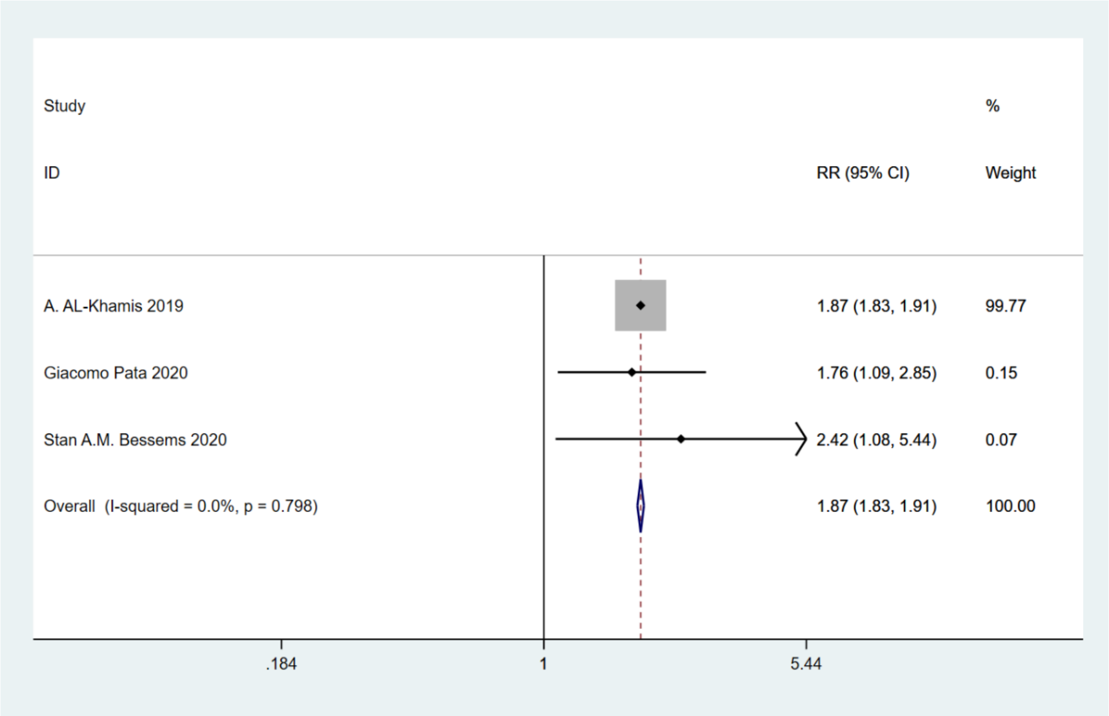

Figure 12: Discharge Destination Not Home

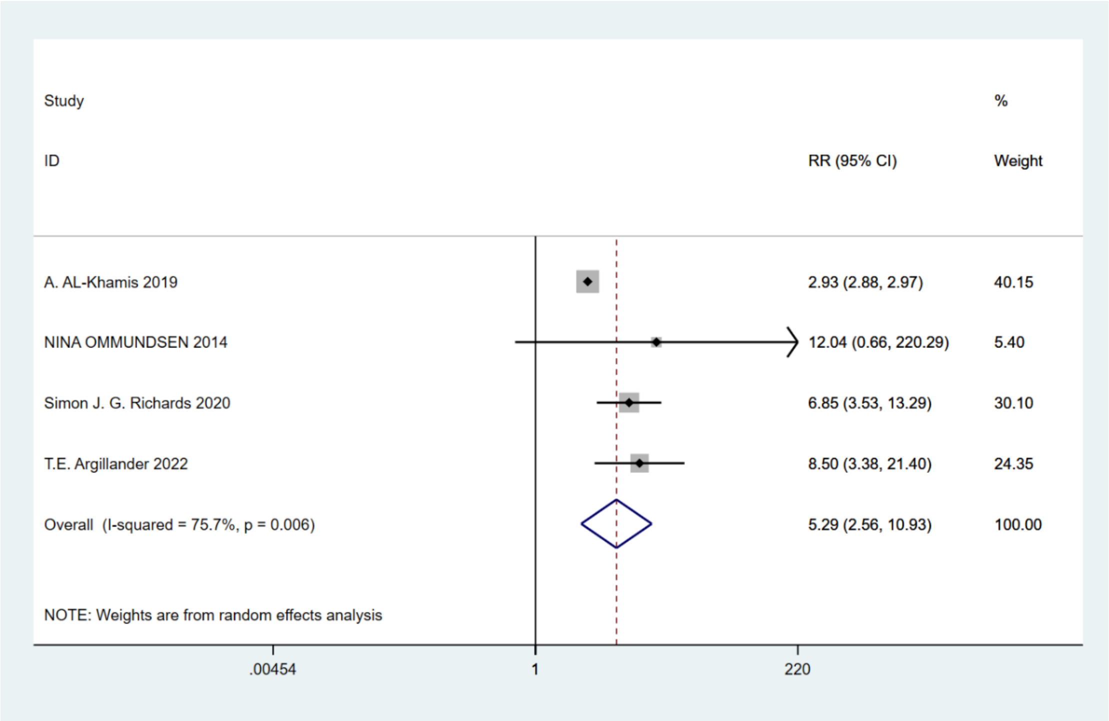


Figure 13: Readmission

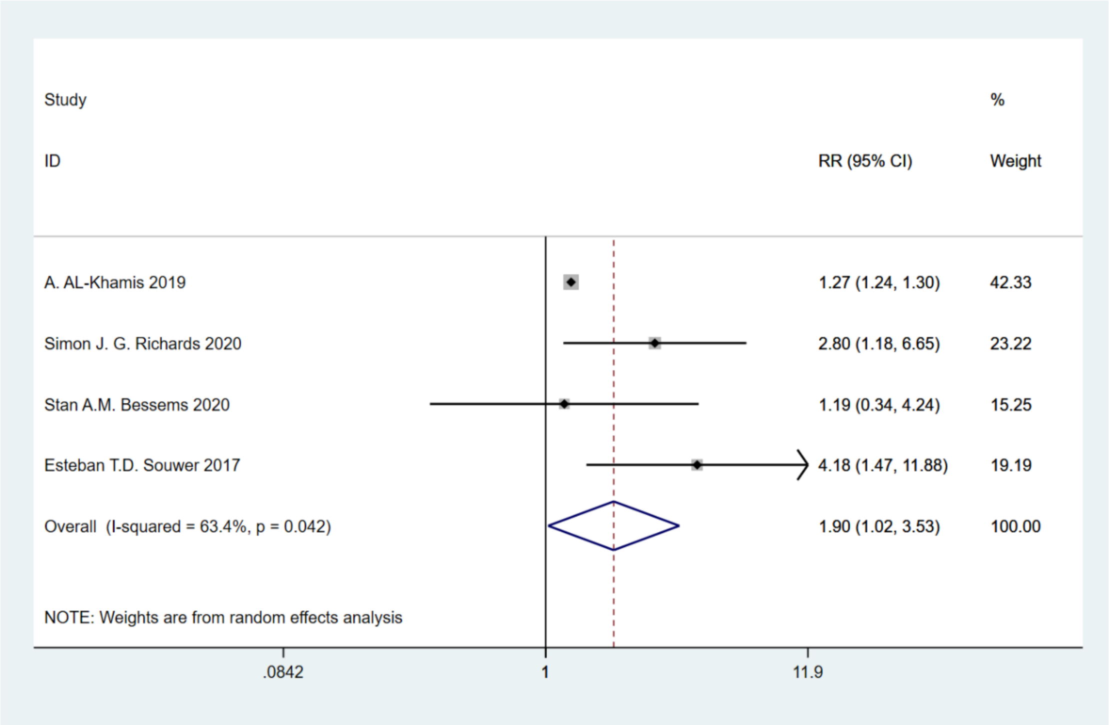

Figure 14: Hospital Stay

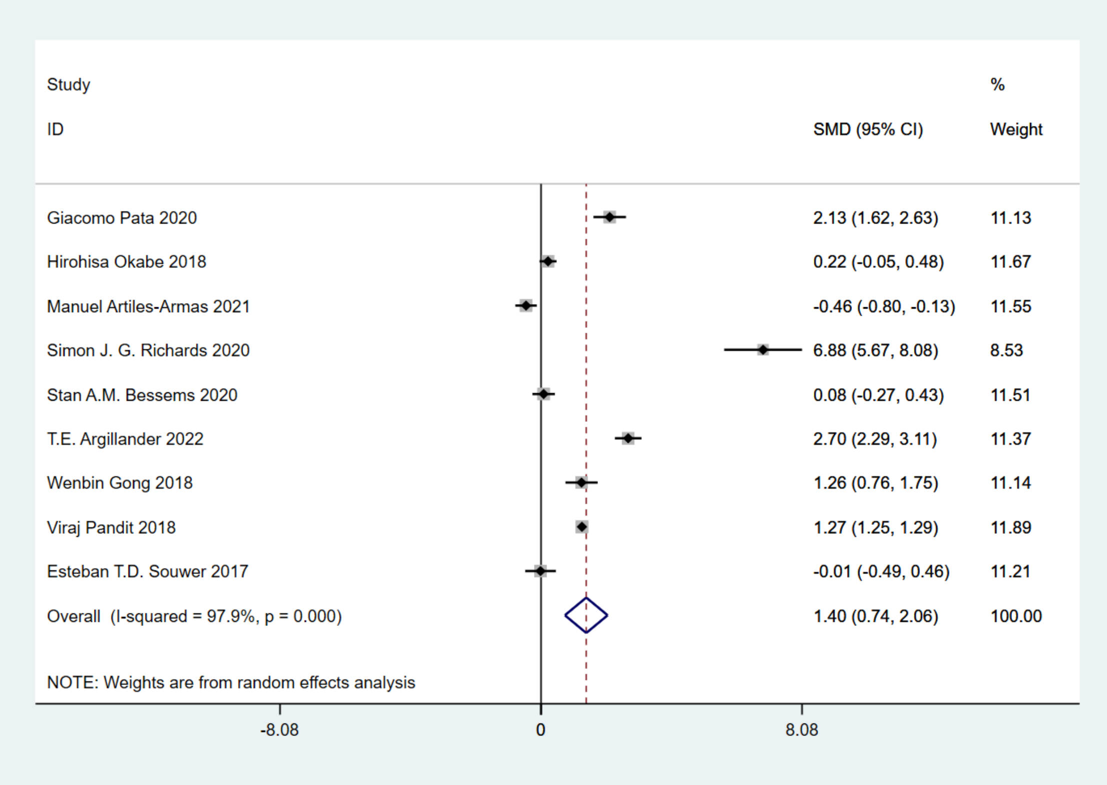

## Subgroup analyses

Figure 15: mortality at different follow-up times

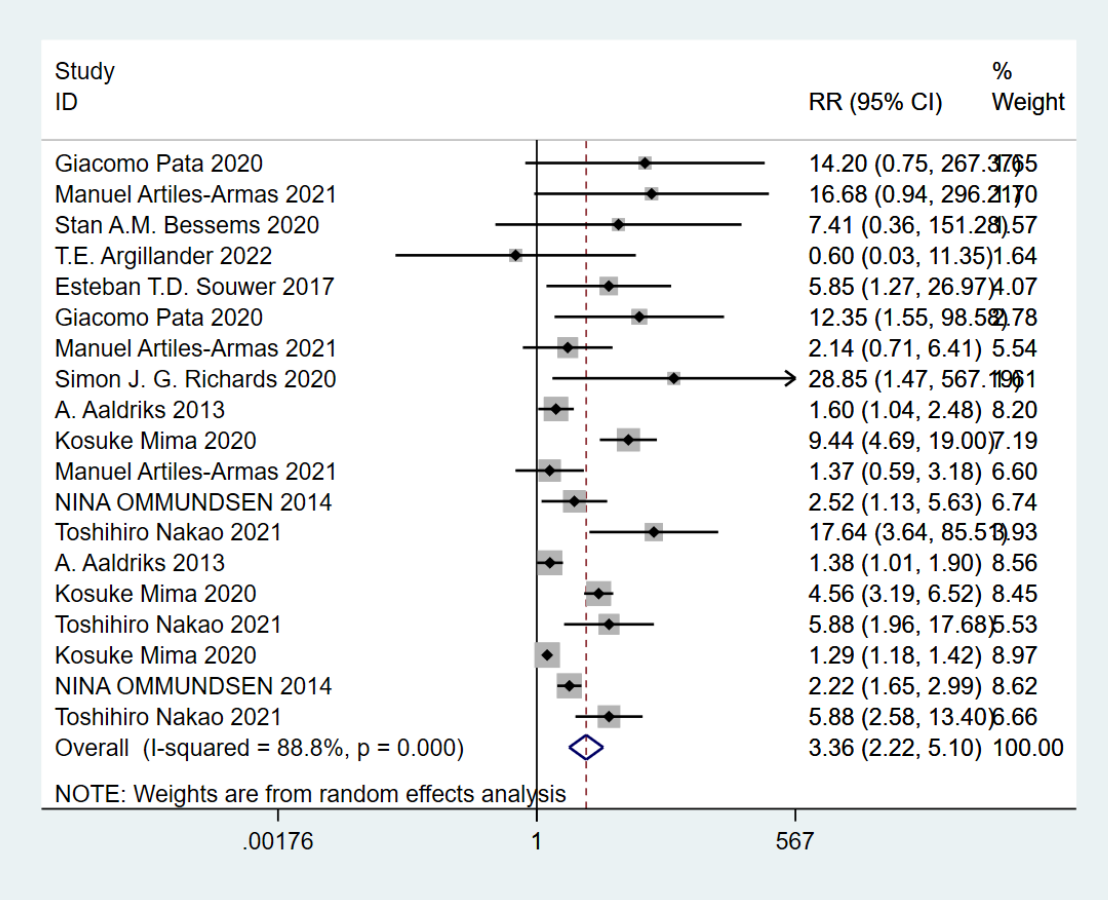

Figure 16: complications of varying degrees after treatment

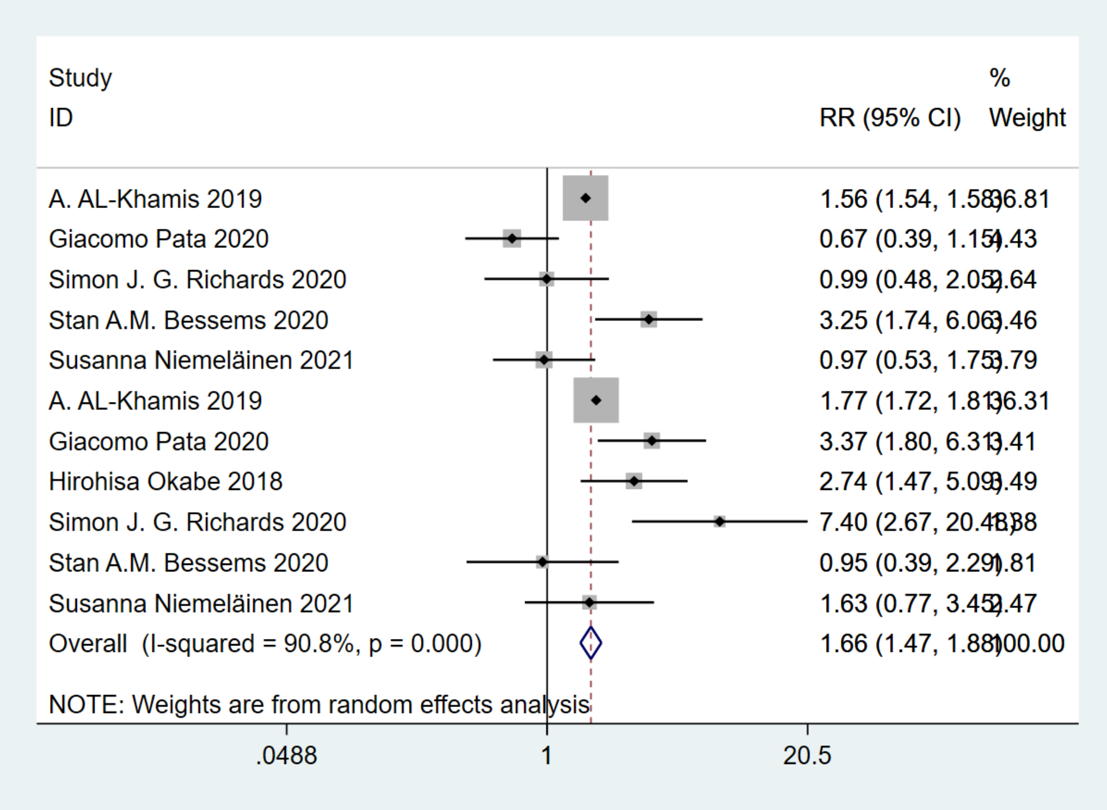


Figure 17

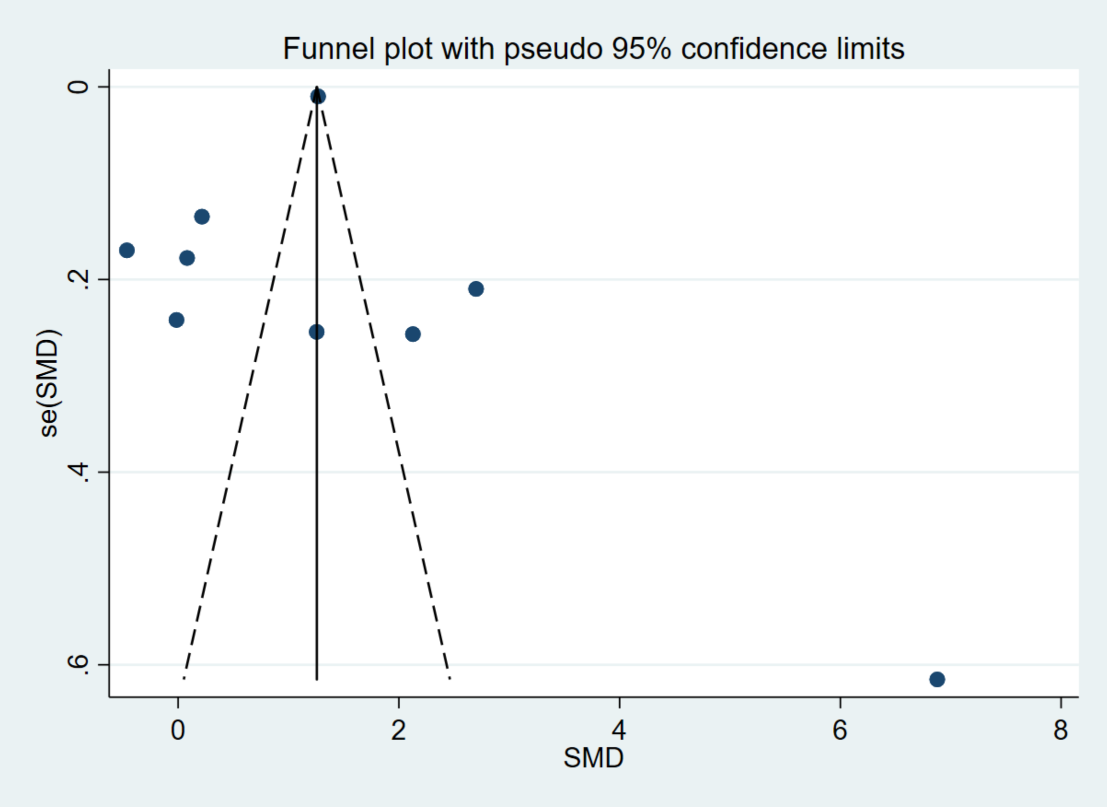

Figure 18

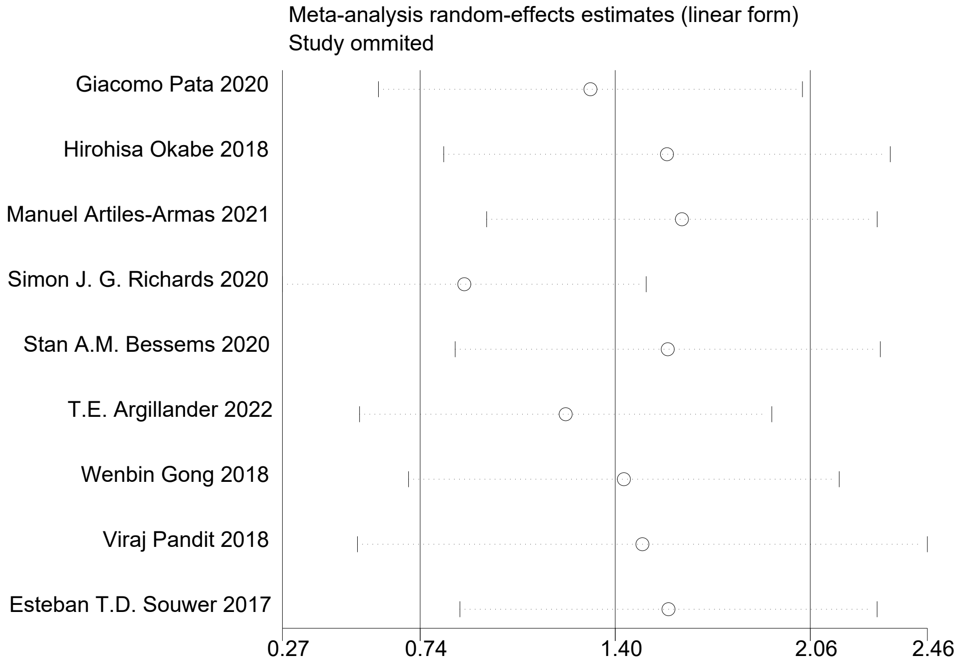


Figure 19

```
Begg's Test

  adj. Kendall's Score (P-Q) =        14
          Std. Dev. of Score =      9.59
           Number of Studies =         9
                           z =      1.46
                    Pr > |z| =     0.144
                           z =      1.36 (continuity corrected)
                    Pr > |z| =     0.175 (continuity corrected)
```

Egger's test

| Std_Eff | Coef. | Std. Err. | t | P>\|t\| | [95% Conf. | Interval] |
|---|---|---|---|---|---|---|
| slope | 1.274233 | .0795048 | 16.03 | 0.000 | 1.086233 | 1.462232 |
| bias | -1.285761 | 2.70172 | -0.48 | 0.649 | -7.674315 | 5.102792 |
