# Supplementary material for: Frailty affects prognosis in patients with colorectal cancer: A systematic review and meta-analysis
Source: Front Oncol. 2022 Nov 3;12:1017183. doi: 10.3389/fonc.2022.1017183 (PMC9669723; doi:10.3389/fonc.2022.1017183)
Supplement: Supplementary file 1 [file DataSheet_1.zip › Table 1.DOCX]

| First author, year | Country | Population | Study design | Assessment of frailty | Frail(n) | Type of treatment | Quality Assessment (Newcastle-Ottawa Scale (NOS)) |
| --- | --- | --- | --- | --- | --- | --- | --- |
| A. Aaldriks,2013 | Netherlands | N=143 41% female Mean age of 75 (range 70-92 years) | prospective study | Groningen Frailty Indicator (GFI) | 34 | Chemotherapy (Adjuvant Chemotherapy,n=54,54%female; Palliative Chemotherapy,n=89,34%female) | 7 |
| A. AL-Khamis,2019 | USA | N=295490 52.5% female Age≥18 years (45.4% ≥65 years) 72.7% White 9.1% Black 2.5% Asian | retrospective study | Five-item modified frailty index (5-mFI) | 53230 | elective or non-elective colorectal procedure(exclude emergency procedure) | 9 |
| Giacomo Pata,2020 | Italy | N=104 47% female The median age was 81 years (range 75–95 years) | prospective multicentric cohort study | The Multidimensional Prognostic Index (MPI; Pilotto et al) | 34 | colorectal cancer surgery | 8 |
| Hirohisa Okabe,2018 | Japan | N=269 38% female Age≥65 years | retrospective study | Clinical Frailty Scale (CFS)(Rockwood K et al. A global clinical measure offitness and frailty in elderly people) | 78 | elective colorectal surgery(palliative procedures were excluded from the study) | 8 |
| Koichi Tamura,2021 | Japan | N=500 41.8% female Median age was 76 years(range 65-96 years) 10 patients≥90 years | prospective study | The Kihon Checklist (KCL) (directed by the Japanese Ministry of Health, Labor and Welfare) | 164 | elective colorectal surgery | 7 |
| Kosuke Mima,2020 | Japan | N=729 47% female Age≥18 years (46% ≥75 years) | retrospective study | Clinical Frailty Scale (CFS)(Rockwood K et al. A global clinical measure offitness and frailty in elderly people) | 253 | curative resection of colorectal cancer | 7 |
| Manuel Artiles-Armas,2021 | Spain | N=149 35.6% female Median age was 75 years (range 72–80 years) | prospective cohort study | The Canadian Study of Health and Aging-Clinical Frailty Scale (CSHA-CFS) | 59 | elective colorectal surgery | 9 |
| NINA OMMUNDSEN,2014 | Norway | N=178 57% female Age≥70 years (6% ≥90 years) | prospective study | Geriatric assessment (GA)(Ellis G et al,Comprehensive geriatric assessment for older adults admitted to hospital,2011) | 76 | elective surgery | 7 |
| Simon J. G. Richards,2020 | New Zealand | N=86 50% female Median age was 76 years (range 72–81 years) | prospective observational study | The Edmonton Frail Scale (EFS) | 12 | elective colorectal cancer surgery | 8 |
| Stan A.M. Bessems,2020 | Netherlands | N=132 44% female Median age was 78 years (range 70–90 years) | retrospective observational study | the Geriatric-8 (G8) and the 4-m gait speed test (4MGST) | 53 | elective colorectal cancer surgery | 8 |
| T.E. Argillander,2022 | Netherlands | N=231 55% female Median age was 76 years (range 73–81 years) | retrospective cohort study | Groningen Frailty Indicator (GFI) | 44 | colorectal cancer (CRC) surgery | 8 |
| Wenbin Gong,2018 | China | N=241 46.5% female The mean age was 68.4 years (SD 11.7) | retrospective study | the Modified Frailty Index (mFI) (derived from the Canadian Study of Health and Aging Frailty Index) | 19 (mFI:Intermediate,n=81;low,n=141) | elective colorectal cancer resections(Emergency cases and non-primary tumor resections were excluded) | 7 |
| K. Beukers,2021 | Netherlands | N=97 51.5% female The mean age was 77.2 years (SD 4.8) | retrospective multicentre study | the Geriatric-8 (G8) | 49 | adjuvant chemotherapy | 6 |
| Susanna Niemeläinen,2021 | Finland | N=161 60% female The mean age was 84.5 years (range 80-97 years) | prospective, multicentre observational study | Clinical Frailty Scale (CFS)(Rockwood K et al. A global clinical measure offitness and frailty in elderly people) | 43 | elective colon cancer surgery | 7 |
| Viraj Pandit,2018 | USA | N=53652 38% female The mean age was 69 years (SD 19)  40% White | retrospective study | CCFI（Seven variables assessed in the Canadian Study of Health and Aging Frailty Index  (CSHA-FI) were matched to preoperative variables collected in the NIS database） | 18241 | elective colon cancer surgery(excluded patients who underwent emergent  surgery or had rectal cancer) | 8 |
| Esteban T.D. Souwer,2017 | Netherlands | N=139 45% female The mean age was 77.7 years (range 75.0–82.8 years) | prospective cohort study | the Geriatric 8 (G8) and Identification of Seniors at Risk for Hospitalized Patients (ISAR-HP) | 20 | colorectal cancer surgery(exclude emergency surgery , Transanal Endoscopic Microsurgery, stage IV disease and synchronous cancer at time of diagnosis) | 8 |
| Elizabeth M. Cespedes Feliciano,2020 | USA | N=126 100% female | multicenter, prospective cohort study | a frailty score( defined in Woods NF et al 2005;Fried LP et al 2001;Erratum.J Am Geriatr Soc. 2017) | 78 | Not mentioned | 7 |
| Toshihiro Nakao,2021 | Japan | N=108 33.3% female Median age was 70 years (range 42–93 years) | retrospective study | Clinical Frailty Scale (CFS)(Rockwood K et al. A global clinical measure offitness and frailty in elderly people) | 11 | colorectal cancer radical surgery | 8 |
