# Supplementary material for: Frailty affects prognosis in patients with colorectal cancer: A systematic review and meta-analysis
Source: Front Oncol. 2022 Nov 3;12:1017183. doi: 10.3389/fonc.2022.1017183 (PMC9669723; doi:10.3389/fonc.2022.1017183)
Supplement: Supplementary file 1 [file DataSheet_1.zip › Table 2.DOCX]

|  | | I-squared | p | RR (95%CI) | Number of studies |
| --- | --- | --- | --- | --- | --- |
| Mortality | | 88.8% | 0 | 3.36 | 12 |
| Complications | | 90.8% | 0 | 1.66 | 12 |
| Delirium | | 25.5 | 0.261 | 3.08 | 3 |
| Postoperative blood transfusion | | 0.0 | 0.798 | 1.87 | 3 |
| Discharge destination not home | | 75.7 | 0.006 | 5.29 | 4 |
| Readmission | | 63.4 | 0.042 | 1.90 | 4 |
| Hospital stay | | 97.9 | 0 | 1.40 | 9 |
| Subgroup analysis | | | | | |
| Complications | minor complications | 78.8% | 0.001 | 1.28 | 5 |
|  | severe complications | 67.8% | 0.008 | 2.26 | 6 |
| Mortality | 30-day | 0.0% | 0.526 | 5..80 | 5 |
|  | 90-day | 51.6% | 0.127 | 6.17 | 3 |
|  | 1-year | 85.5% | 0 | 3.50 | 5 |
|  | 2-year | 93.3% | 0 | 3.15 | 3 |
|  | 5-year | 92.0% | 0 | 2.26 | 3 |

***Table 2***
