# Supplementary figures and images for: Frailty affects prognosis in patients with colorectal cancer: A systematic review and meta-analysis
Source: Front Oncol. 2022 Nov 3;12:1017183. doi: 10.3389/fonc.2022.1017183 (PMC9669723; doi:10.3389/fonc.2022.1017183)

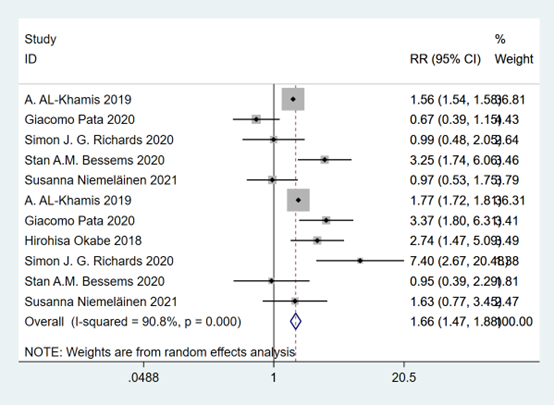

Supplement: Supplementary file 1 [file DataSheet_1.zip › Images/Image 1.TIF]

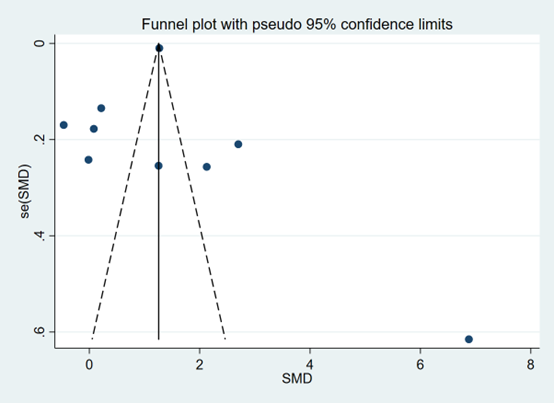

Supplement: Supplementary file 1 [file DataSheet_1.zip › Images/Image 2.TIF]

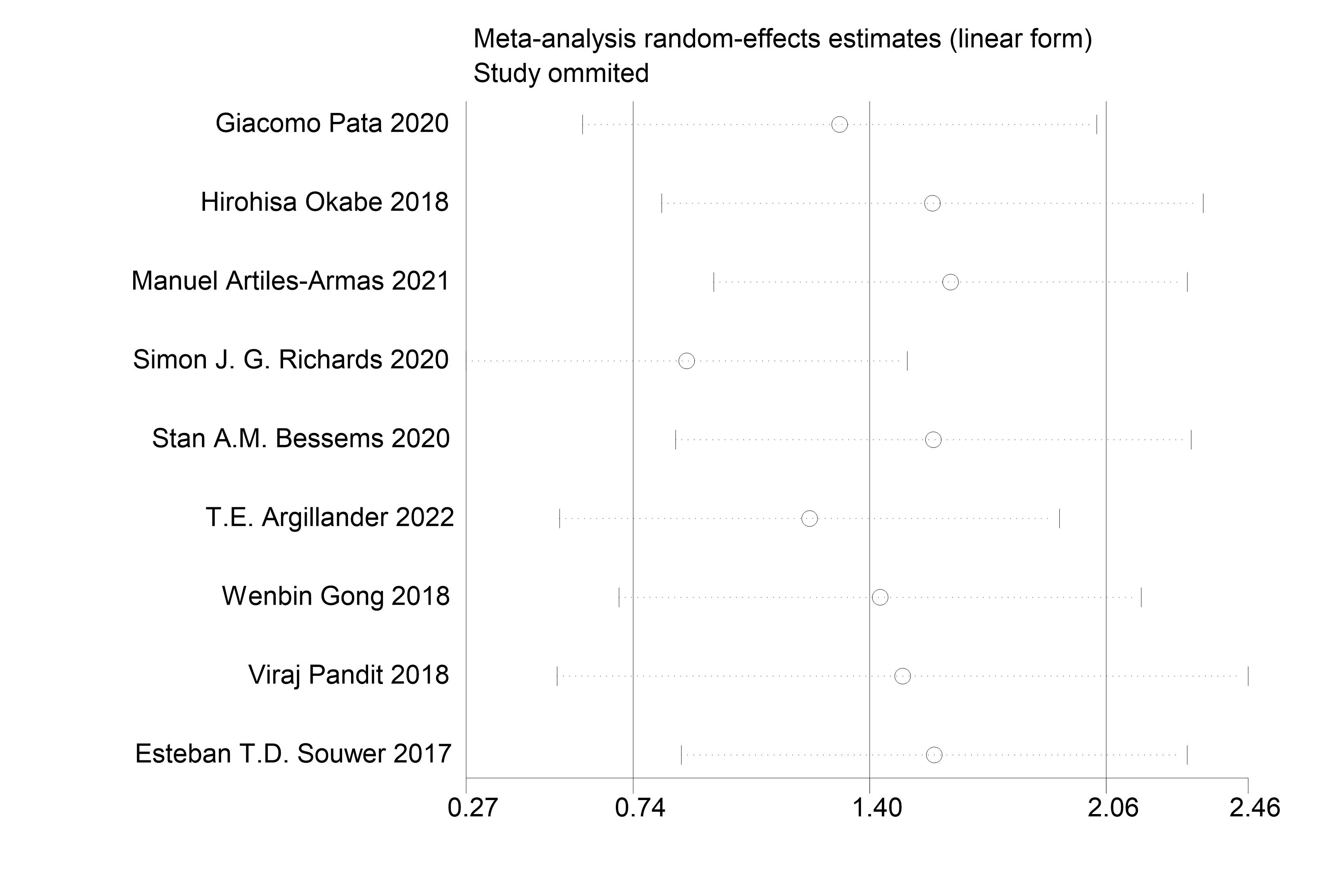

Supplement: Supplementary file 1 [file DataSheet_1.zip › Images/Image 3.TIF]

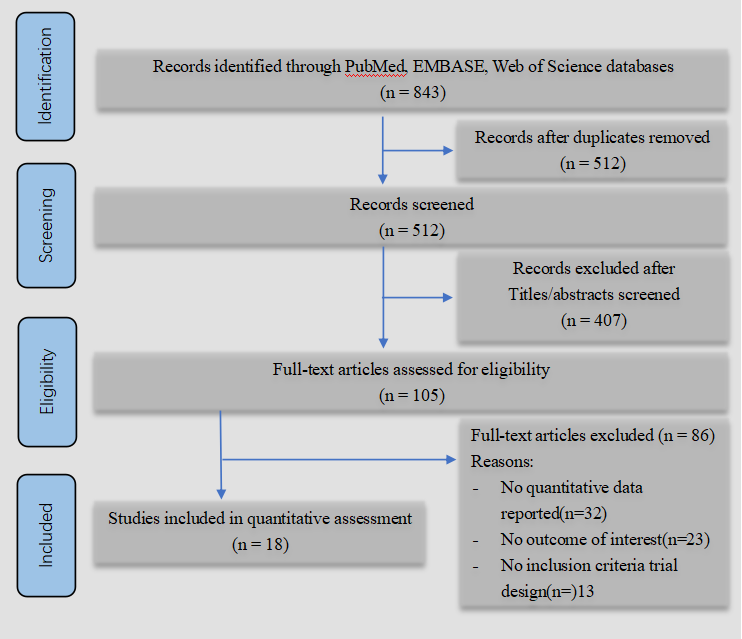

Supplement: Supplementary file 1 [file DataSheet_1.zip › Images/Image 4.PNG]

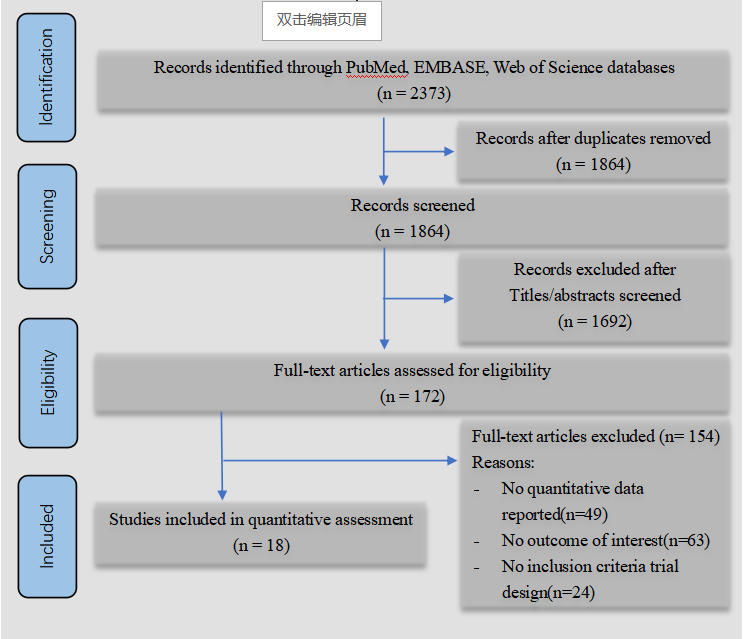

Supplement: Supplementary file 1 [file DataSheet_1.zip › Images/Image 5.PNG]
